# Active Healing of Microtubule-Motor Networks

**Published:** 2025-05-22

**Authors:** Fan Yang, Shichen Liu, Hao Wang, Heun Jin Lee, Rob Phillips, Matt Thomson

**Affiliations:** 1 Division of Biology and Biological Engineering, California Institute of Technology, Pasadena, CA, USA; 2 Department of Applied Physics, California Institute of Technology, Pasadena, CA, USA

## Abstract

Cytoskeletal networks have a self-healing property where networks can repair defects to maintain structural integrity. However, both the mechanisms and dynamics of healing remain largely unknown. Here we report a healing mechanism in microtubule-motor networks by active crosslinking. We directly generate defects using a light-controlled microtubule-motor system in O-shaped and V-shaped networks, and observe that the defects can self-heal. Combining theory and experiment, we find that the V-shaped networks must overcome internal elastic resistance in order to heal cracks, giving rise to a bifurcation of dynamics dependent on the initial opening angle of the crack: the crack merges below a critical angle and opens up at larger angles. Simulation of a continuum model reproduces the bifurcation dynamics, revealing the importance of a boundary layer where free motors and microtubules can actively crosslink and thereby heal the defects. We also formulate a simple elastic-rod model that can qualitatively predict the critical angle, which is found to be tunable by the network geometry.

Cytoskeletal networks can dynamically reconfigure themselves and generate force to fulfill crucial functions in life, such as mechanical support, motility and division of cells. Furthermore, in neurons, microtubules are organized into parallel arrays that serve as tracks for cargo transport [1]. After an axon is injured, the rearrangement of microtubule orientations into parallel arrays plays a key role in axon regeneration [2]. Kinesin motors that can actively bind and walk on microtubules may contribute to the healing and alignment of microtubule networks [3]. However, such mechanisms of healing by motors have not been studied. Past research on self-healing cytoskeletal networks mainly focuses on mechanisms through adding or reassembling the subunits that make up the cytoskeleton. For example, individual microtubules are found to be capable of incorporating free tubulins to repair lattice defects [4]. At the network level, filamentous actin hydrogels can restore their storage modulus through dynamic polymerization and depolymerization of globular actin, after a shear strain is removed [5]. Motor proteins can also reconnect laser-ablated microtubule bundles in mitotic spindles [6]. In this Letter, we investigate an underexplored self-healing mechanism, where active crosslinking and diffusion by motor proteins can heal defects in microtubule networks. In particular, we focus on healing through the closure of defects—such as cuts in O-shaped networks and cracks in V-shaped networks.

Our reconstituted microtubule-motor system [7, 8] provides a light-controllable platform with minimum components that can generate self-healing networks and elucidate the underlying mechanisms. The experimental system [8] consists of free microtubules, light-activatable motor proteins, ATP and buffer solutions, placed in a flow cell, whose height, around 100 *μ*m, is much smaller than its horizontal dimensions (Supplemental Material). Depolymerization and polymerization of tubulins can be neglected in our experiments. The microtubules are stabilized to minimize depolymerization [7]. Polymerization doubles the average microtubule length, initially around 1.3 *μm*, every 4 hours, which is very slow compared to the healing dynamics at the scale of minutes. The engineered motor proteins can “link” under blue light. We use the terms “linked” and “unlinked” motors to distinguish these two states. Microtubule networks of arbitrary shapes can be generated through light projections onto the flow cell. The networks are contractile due to crosslinking by motors.

We directly generate O-shaped networks with defects to investigate whether they can self-heal. As shown in Fig. 1(a–c), we find that when the gap width b is small, the defect can heal and the O-shaped network contracts as a whole. In contrast, when b is large, the defect expands further, leading to the opening up of the O-shaped network. The healing dynamics—either opening or closing of the gap—is decoupled from the overall contraction of the network. The healing success rates, defined by the percentage of successful healing experiments among total experimental replicates, are documented in Fig. 1(d) with varying geometrical parameters. Our experiments reveal a consistent critical gap threshold $b_{c}$, within the range of 5–10 *μ*m, that governs the self-healing behavior of O-shaped networks. Across different inner and outer radii, the O-shaped network tends to heal successfully when $b<b_{c}$ and fail to heal when $b>b_{c}$.

The critical gap threshold $b_{c}$ can be quantitatively predicted based on a diffusive boundary layer of free linked motors that form next to the defect interfaces. The motors are initially linked within the light region and dynamically crosslink microtubules by constantly binding on and detaching from the network. We hypothesize that there is a layer of free linked motors at the network surface due to diffusion of motors that detach from the network. We can estimate the layer thickness h by balancing two fluxes: the unbinding flux of motors $k^{\text{off}}d_{b}$, where $k^{\text{off}}$ is the unbinding rate and $d_{b}$ is the crosslinked motor concentration, and the diffusive flux of free motors $D_{d}\nabla^{2}d_{f}$, with diffusivity $D_{d}$ and the free motor concentration $d_{f}$. Balancing these two fluxes yields a boundary layer thickness $h∼\sqrt{D_{d}d_{f}/k^{\text{off}}d_{b}}$. Within the boundary layer, the ratio $d_{f}/d_{b}$ ranges from much greater than 1 on the side of the crosslinked network, to much smaller than 1 on the side facing the free solution. As an order-of-magnitude estimate for h, we can assume $d_{f}/d_{b}∼1$. Further using $D_{d}=20\mu {\text{m}}^{2}/\text{s}$ [9] and $k^{\text{off}}=0.6\;{\text{s}}^{-1}$ [10, 11], the boundary layer thickness is found to be h∼6μm. In the O-ring experiments, the critical gap threshould $b_{c}$ should satisfy $b_{c}\approx h$ to enable overlap of the boundary layers at the two opposing defect interfaces. Therefore, our estimate h∼6μm is in quantitative agreement with the experimental observed range of $b_{c}$ between 5−10μm, demonstrating that the overlap of motor boundary layers may be critical for successful healing.

To further test the boundary-layer hypothesis, we create V-shaped networks to mimic cracks, and find that there exists a critical initial opening angle above which the network buckles, and below which it merges. Fig. 2(a) and 2(b) show two networks with the same initial arm lengths and widths but different opening angles. The network with the larger angle in Fig. 2(a) keeps opening up as it contracts. Its two arms bend outwards and form a convex shape. In contrast, the network with the smaller initial angle in Fig. 2(b) closes in and the two arms zip up, forming a concave shape. The critical opening angle also depends on the network geometry. We generate two networks with fixed arm lengths and opening angles but different widths, as shown in Fig. 2(c) and 2(d), and find that the thinner network buckles outwards while the thicker one bends inwards, indicating that the critical angle can be tuned by the arm shape.

The two distinct phenomena in Fig. 2(a) and 2(b) demonstrate a bifurcation of the active network dynamics dependent on the initial opening angles. We denote the dynamics in Fig. 2(a) and Fig. 2(b) the buckling-dominated and merging-dominated regimes, respectively. The two regimes can be differentiated quantitatively by curvature of the network. Given a centerline profile y(x) (inset in Fig. 3), the local curvature κ is defined as $\kappa =y^{\prime \prime }/{\left(1+y^{\prime 2}\right)}^{3/2}$. We define the mean curvature $\left[\kappa \right]$ along the centerline as $\left[\kappa \right]=\int_{x_{0}}^{x_{t}}\frac{y^{\prime \prime }}{1+y^{\prime 2}}\text{d}x/\int_{x_{0}}^{x_{t}}\sqrt{1+y^{\prime 2}}\text{d}x$, where $x_{0}$ and $x_{t}$ are the x-coordinates of the starting and ending points of the centerline, respectively. Time evolutions of mean curvatures in Fig. 2(a) and 2(b) are plotted in Fig. 3. By our definition, a negative curvature represents a concave shape, which emerges in the merging-dominated regime, such as Fig. 2(b). More experimental images of concave networks can be found in Fig. S6 in the Supplemental Material. In contrast, a positive curvature, that is, a convex shape, arises in the buckling-dominated regime, where the two arms bend outwards and away from each other, as in Fig. 2(a).

We use simulations based on a field theory model to uncover mechanisms of self-healing. The simulations in Fig. 2 are based on a three-phase model from our previous work [8] (Supplemental Material) The simulation can reproduce the bifurcation dynamics (Fig. 2) and the simulated curvatures are in qualitative agreement with experiments (Fig. 3]). A key step in our simulations is to explicitly model the process of active crosslinking of free microtubules and linked motors into the crosslinked network, that is

(1) $$\frac{\partial \bar{c}}{\partial \bar{t}}+\bar{\nabla }\cdot (\bar{cv})={\bar{p}}_{1}^{\text{on}}{\bar{c}}_{f}{\bar{d}}_{f}-{\bar{p}}_{1}^{\text{off}}\bar{c},$$

where $\bar{c}\left({\bar{c}}_{f}\right)$ is the crosslinked (free) microtubule concentration, ${\bar{d}}_{f}$ is the free linked motor concentration, $\bar{t}$ is time, $\bar{v}$ is the crosslinked microtubule velocity, ${\bar{p}}_{1}^{\text{on}}$ and ${\bar{p}}_{1}^{\text{off}}$ are the crosslinking and uncrosslinking rates, respectively. Our simulations are non-dimensionalized (Supplemental Material) and the symbols with an overbar represent dimensionless variables. To demonstrate that the merging is induced by active crosslinking, we simulate the bifurcation diagram of the network in Fig. 2(d) by varying the crosslinking rates ${\bar{p}}_{1}^{\text{on}}$ and documenting whether the network buckles or merges under different initial crack angles θ. The result is in Fig. 4, showing that increasing the crosslinking rate renders the network easier to merge and therefore confirming that the merging process is induced by active crosslinking.

The boundary-layer hypothesis can explain the opening-angle dependency of the bifurcation. The overlapped region expands with decreasing opening angle θ, and therefore merging should prevail at small θ. When the network is merging-dominated, the overlapped region becomes a “ zipping front” that can propagate along and zip up the two network arms. It is well known that an elastic rod under compression can buckle when the compressive load exceeds a threshold. Buckling has also been reported in rectangular active networks [7]. The V-shaped network is a joint of two rectangular networks, where buckling can take place. Therefore, we propose that the opening-up dynamics observed in Fig. 2(a) and 2(c) is a result of the buckling instability induced by the compressive active stress. And when buckling takes place, the two arms will only bend outwards in order to reduce the bending energy, which is quadratic in the local curvature [12], at the kink.

Based on the boundary layer and buckling hypotheses, we propose a simple elastic-rod model to predict the critical angle in bifurcation. We assume the crosslinked network is an elastic rod, as shown in Fig. 5. The active stress is assumed to be $\sigma_{a}$, generating a compressive force in each arm that is $F_{a}=\sigma_{a}wd$, with w and d the arm width and depth, respectively. Inside the overlapped boundary layer, active crosslinking introduces an attractive force $F_{h}$ between the two network arms. We assume the surface force density is σ (inset in Fig. 5), and the attractive force on each arm is then $F_{h}=\sigma dh/\sin\theta$, where θ is the opening angle and h is the boundary layer width. The tangential component of $F_{h}$ along the network arm introduces a tension and the minimum compression within each arm is $F_{c}\approx \sigma_{a}wd-\frac{\sigma dh}{2\sin(\theta /2)}$. The bifurcation of network dynamics is governed by two competing mechanisms, one towards buckling, characterized by $F_{c}$, and the other towards merging, characterized by $F_{h}$. We use the classical Euler’s buckling load $F_{b}=\pi^{2}EI/l^{2}$ [13] to approximate the critical load to buckle each network arm, where E is the Young’s modulus, $I=dw^{3}/12$ is the moment of inertia. Therefore, the critical angle $\theta^{*}$ is determined by $F_{c}=F_{b}$, and it follows that

(2) $$\theta^{*}=2\arcsin\frac{\sigma h}{2w\left(\sigma_{a}-CE\delta^{2}\right)},$$

where δ=w/l is the aspect ratio of each arm and $C=\pi^{2}/12$ is a constant. The network will heal when $\theta <\theta^{*}$ and buckle when $\theta >\theta^{*}$. From (2) we can see that $\theta^{*}$ can be tuned by two dimensionless geometric parameters, the ratio of the boundary layer width and the arm width h/w, and the aspect ratio of the network δ=w/l. The network shape w and δ are programmable through light signals in experiments and simulations. Fixing δ and increasing w will decrease the critical angle $\theta^{*}$. This is because the healing force $F_{h}$ is only determined by θ and does not depend on w and δ, and the compression $F_{c}$ in each arm is linear in w. As the arm width grows, the critical angle needs to shrink in order to generate a greater overlap region, and consequently a larger $F_{h}$, to overcome the increasing compression. Fixing w and decreasing the aspect ratio δ will also decrease $\theta^{*}$. The compression $F_{c}$ also does not vary with δ in this case. However, decreasing δ will make the network more elongated and easier to buckle. Therefore, a smaller critical angle is required to generate a larger attractive force $F_{h}$ in order to overcome the elastic instability. We note that there is always overlapping of boundary layers in the tip region and thus the tip region is always concave. The convexity and concavity predicted by our simple model, as described by equation (2), applies only to the bulk region away from the tip.

To test how the network geometry can tune the bifurcation dynamics, we run simulations with different opening angles θ and vary the network width w and aspect ratio δ. Whether the network buckles or merges in each simulation is documented in the bifurcation diagrams in Fig. 6. We first fix δ=0.1 and simulate networks with different w. The bifurcation phase diagram and the theoretical prediction (2) are plotted in Fig. 4(a). As the network width increases, the critical angle $\theta^{*}$ decreases. This confirms our theory (2) that increasing the size of the network while preserving the shape will make buckling easier and merging more difficult. This is because the overlapped boundary layer and the healing force $F_{h}$ do not scale with the network size while the active compression $F_{c}$ is proportional to w. As the network dimension increases, the merging effect becomes less significant. Similarly, the bifurcation phase diagram (Fig. 6(b)) with different δ and fixed w is also consistent with our theory. As the aspect ratio δ increases, the network arms become shorter and thus more difficult to buckle. Both simulated phase diagrams are in quantitative agreement with experimental data. In general, h, σ, $\sigma_{a}$ and E in our theory (2) are all functions of the local microtubule and motor concentrations. We treat them as constants in plotting the theory in Fig. 6 for simplicity. Even so, the elasti-crod model can qualitatively predict the bifurcation phase diagram and offers a clear explanation for its dependence on w and δ. Furthermore, we can define two dimensionless groups, $A=2w\sigma_{a}/\sigma h$ and $B=2CE\delta^{2}w/\sigma h$, and rewrite equation (2) as $\theta^{*}=2\arcsin\left[{\left(A-B\right)}^{-1}\right]$, where A is the ratio of the active compression and merging forces, and B is the ratio of the critical buckling load and the merging force. Increasing A or decreasing B renders the network easier to buckle and results in a smaller $\theta^{*}$. As an anonymous referee pointed out, the quantitative discrepancy between our simple model and the linear dependence of $\theta^{*}$ on δ predicted by simulations in Fig. 6b may also arise from the omission of effective surface tension in equation (2). This surface tension, arising from the motor activity, may cause the active network to bend inwards to reduce surface area [14]–conceptually analogous to the barreling instability described in Ref. [15].

In summary, we show that the overlapping of motor boundary layers is critical in defect healing of active networks. For V-shaped cracks, the active crosslinking also needs to overcome an elastic instability which will open up the crack, leading to a bifurcation of merging and buckling that can be tuned by the initial network geometry. We also highlight two distinct features of active healing—energy consumption and active stresses—that require further study. Energy consumption is essential for the active binding of motors to microtubules and is a key driver of the healing process, as simulated in Section I.7 of the Supplemental Material. Active stresses, depending on the network geometry, can either promote or hinder healing, offer tunability in active systems. It has been increasingly evident that cytoskeletal networks are gel-like materials [16] and vulnerable to a plethora of mechanical instabilities from self-generated active forces. For example, a bifurcation of in-plane bending and out-of-plane buckling instabilities has been observed in extensile active sheets [17]. Instabilities are not always obstructive. Cells can control and harness instabilities to create useful structures, such as mitotic spindles which are shaped by a barreling-type instability [15]. More work is needed to complete a mechanical-instability phase diagram of active networks and to uncover the regulatory mechanisms used by cells to control such instabilities.

## Supplementary Material

Supplement 1

## Figures and Tables

**FIG. 1.**
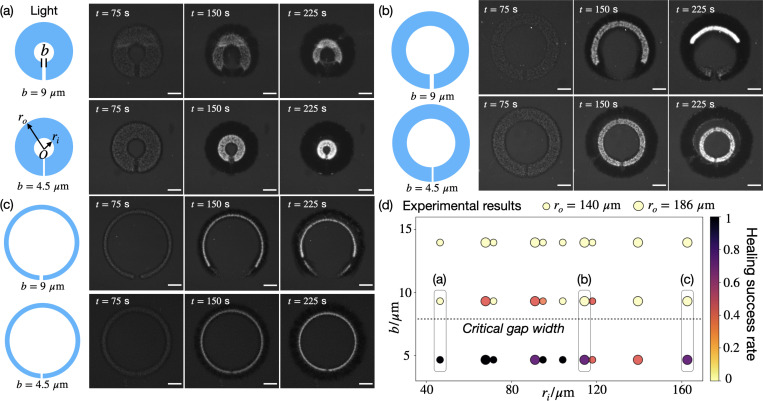
Self-healing behavior of O-shaped active networks is governed by a critical gap width. (a-c) Experimental images of O-shaped networks with gap defects. In each panel, the inner and outer radii are fixed, while two initial gap widths, b=4.5μm and 9 *μ*m, are shown. Scale bars, 200 *μ*m. (d) Experimental measurements of healing success rates with varying gap widths b and inner radii $r_{i}$. Small and big markers correspond to outer radii $r_{o}=140\;\mu \text{m}$ and 186 *μ*m, respectively. Experiments (a-c) are labeled on the diagram.

**FIG. 2.**
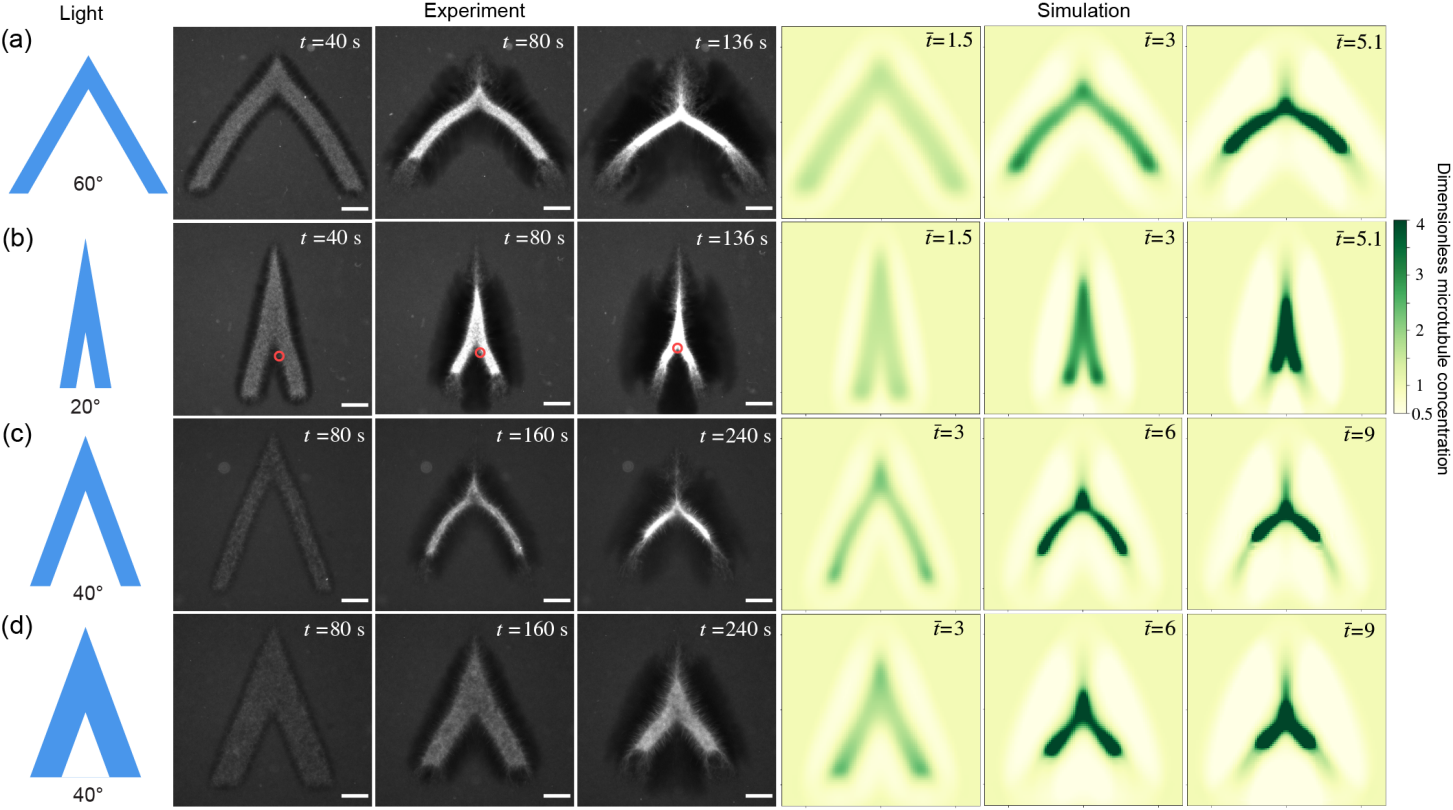
Experiments and simulations of V-shaped active networks show a bifurcation of merging- and buckling-dominated dynamics dependent on the network geometry. (a) and (b) are two networks with the same initial arm lengths and widths but different opening angles. The networks buckle at the large opening angle (a) while merge at the small angle (b). Red circles in (b) track a small protrusion on the right arm which eventually merges with the left arm, demonstrating the partial closure of the crack. (c) and (d) are two networks of the same arm lengths and opening angles but different arm widths. The thinner network (c) buckles outwards while the thicker one (d) bends inwards. The spatiotemporal dimensions of the simulated and experimental images are matched. Simulation details are in the Supplemental Material. $t(\bar{t})$ is (dimensionless) time after the first light pulse. In simulations, the microtubule concentration is non-dimensionalized by the initial microtubule concentration. Scale bar, 100 *μ*m.

**FIG. 3.**
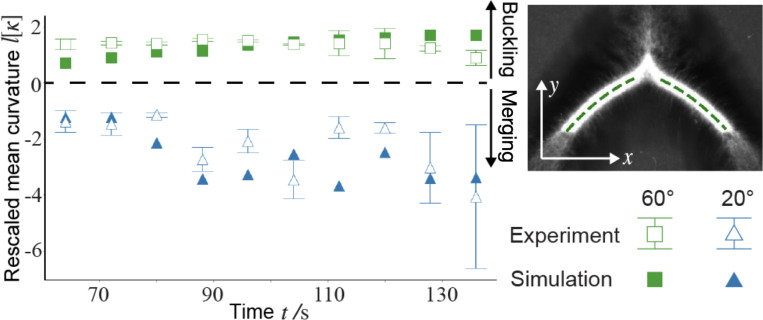
Merging and buckling-dominated dynamics can be differentiated by the network curvature. The curvature is negative (concave) for the former and positive (convex) for the latter. The mean curvature [κ] is averaged over the centerline of each arm excluding the tip region (dotted lines in the upper right inset), and rescaled by the initial arm length l. Error bars represent the difference between left and right arms in a single experiment.

**FIG. 4.**
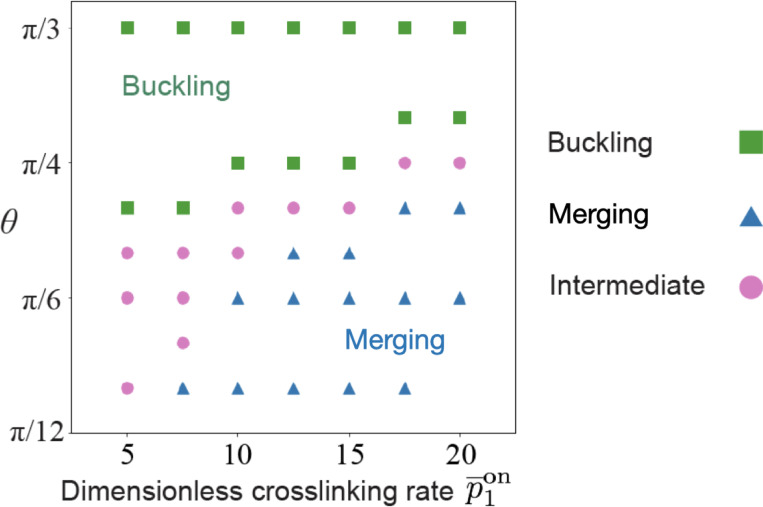
Simulated bifurcation diagram shows that the critical angle increases with the crosslinking rate. The diagram documents whether the network in Fig. 2(d) merges or buckles with varying crosslinking rates and initial crack angles. “Intermediate” represents when the network does not buckle and also does not show significant merging, such as Fig. 2(d). In simulations, the “Intermediate” state is characterized by the average curvature close to 0.

**FIG. 5.**
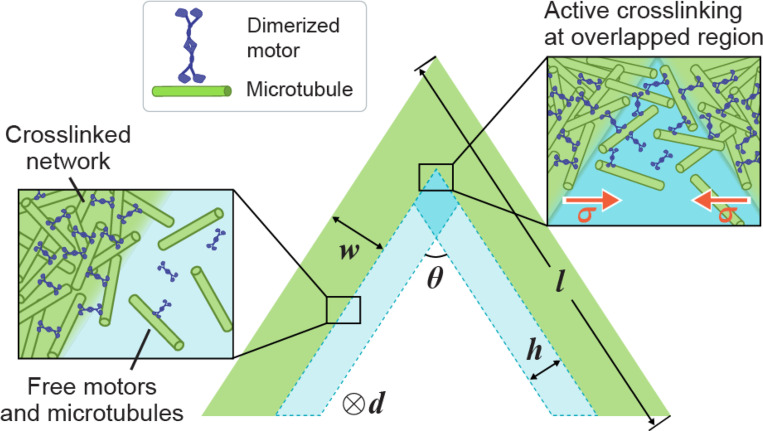
Schematic of the elastic-rod model. The crosslinked network is modeled as a kinked elastic rod (green). There are boundary layers (light blue) of free motors and microtubules next to the network surfaces. Active crosslinking takes place at the overlapped region (dark blue) of the two boundary layers.

**FIG. 6.**
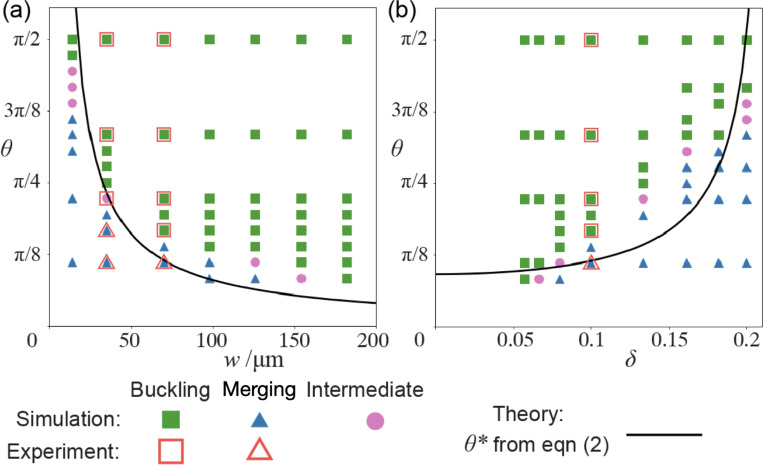
Bifurcation phase diagrams can be qualitatively predicted by the elastic-rod model. We fix δ=0.1 in (a) and w=70μm in (b). The fitting parameters used to plot the theoretical curves are $\sigma h/\sigma_{a}=20\;\mu \text{m}$ and σh/CE=1μm.
