# Geometry-Dependent Defect Merging Induces Bifurcated Dynamics in Active Networks

**Published:** 2025-09-08

**Authors:** Fan Yang, Shichen Liu, Hao Wang, Heun Jin Lee, Rob Phillips, Matt Thomson

**Affiliations:** 1 Division of Biology and Biological Engineering, California Institute of Technology, Pasadena, CA, USA; 2 Department of Applied Physics, California Institute of Technology, Pasadena, CA, USA

## Abstract

Cytoskeletal networks can repair defects to maintain structural integrity. However, the mechanisms and dynamics of defect merging remain poorly understood. Here we report a geometry-tunable merging mechanism in microtubule-motor networks initiated by active crosslinking. We directly generate defects using a light-controlled microtubule-motor system in O-shaped and V-shaped networks, and observe that the defects can self-close. Combining theory and experiment, we find that the V-shaped networks must overcome internal elastic resistance in order to zip up cracks, giving rise to a bifurcation of dynamics dependent on the initial opening angle of the crack: the crack merges below a critical angle and opens up at larger angles. Simulation of a continuum model reproduces the bifurcation dynamics, revealing the importance of overlapping boundary layers where free motors and microtubules can actively crosslink and thereby merge the defects. We also formulate a simple elastic-rod model that can qualitatively predict the critical angle, which is tunable by the network geometry.

Cytoskeletal networks can dynamically reconfigure themselves and generate force to fulfill crucial functions in life, such as mechanical support, motility, and division of cells. Furthermore, in neurons, microtubules are organized into parallel arrays that serve as tracks for cargo transport [1]. After an axon is injured, the rearrangement of microtubule orientations into parallel arrays plays a key role in axon regeneration [2]. Kinesin motors that can actively bind and walk on microtubules may contribute to the healing and alignment of microtubule networks [3]. However, such mechanisms of healing by motors are not well studied. Previous research on self-healing cytoskeletal networks has mainly focused on mechanisms through adding or reassembling the subunits that make up the cytoskeleton. For example, individual microtubules are found to be capable of incorporating free tubulins to repair lattice defects [4]. At the network level, filamentous actin hydrogels can restore their storage modulus through dynamic polymerization and depolymerization of globular actin, after a shear strain is removed [5]. Motor proteins can also reconnect laser-ablated microtubule bundles in mitotic spindles [6]. In this Letter, we investigate how active crosslinking by motor proteins drives geometry-dependent defect merging in O-shaped and V-shaped microtubule networks, leading to bifurcated dynamics.

Our reconstituted microtubule-motor system [7, 8] provides a light-controllable platform with minimum components that can generate self-healing networks and elucidate the underlying mechanisms. The experimental system [8] consists of free microtubules, light-activatable motor proteins, ATP and buffer solutions, placed in a flow cell, whose height, around 100 *μ*m, is much smaller than its horizontal dimensions (Supplemental Material). Depolymerization and polymerization of tubulins can be neglected in our experiments. The microtubules are stabilized to minimize depolymerization [7]. Polymerization doubles the average microtubule length, initially around 1.3 *μm*, every 4 hours, which is very slow compared to the healing dynamics at the scale of minutes. The engineered motor proteins can “link” under blue light. We use the terms “linked” and “unlinked” motors to distinguish these two states. Microtubule networks of arbitrary shapes can be generated through light projections onto the flow cell. The networks are contractile due to crosslinking by motors.

We directly generate O-shaped networks with defects to investigate whether they can self-heal. As shown in Fig. 1(a-c), when the gap width b is small, the defect merges and the O-shaped network contracts as a whole. In contrast, when b is large, the defect expands, leading to the opening of the O-shaped network. The dynamics—either opening or closing of the gap—is decoupled from the overall contraction of the network. The healing success rates, defined as the percentage of successful merging experiments among total experimental replicates, are documented in Fig. 1(d) with varying geometrical parameters. Our experiments reveal a consistent critical gap threshold $b_{c}$, within the range of 13–26 *μ*m, that governs the self-healing behavior of O-shaped networks. Across various inner and outer radii, the O-shaped network tends to merge successfully when $b<b_{c}$ and fail to merge when $b>b_{c}$.

We measure the light intensity across the gap and find that the critical gap threshold, $b_{c}$, is determined by the effective activation region of the projected light. At the edges of the projected light pattern, the light intensity decays to the background level within a ~ 20 *μ*m layer, as shown in Fig. S7 of the Supplemental Material. When the projected gap width is b=13 *μ*m, the two opposing light decay regions strongly overlap, whereas for b=26 *μ*m the overlapping region is small. We hypothesize that these light decay regions give rise to a boundary layer of linked motors adjacent to the defect interfaces. The gap closes only when the boundary layers from opposite sides significantly overlap, allowing the linked motors within the overlapping region to crosslink microtubules and merge the gap.

To further test the boundary-layer hypothesis, we create V-shaped networks to mimic cracks, and find that there exists a critical initial opening angle above which the network buckles, and below which it merges. Fig. 2(a) and 2(b) show two networks with the same initial arm lengths and widths but different opening angles. The network with the larger angle in Fig. 2(a) keeps opening up as it contracts. Its two arms bend outwards and form a convex shape. In contrast, the network with the smaller initial angle in Fig. 2(b) closes in and the two arms zip up, forming a concave shape. The critical opening angle also depends on the network geometry. We generate two networks with fixed arm lengths and opening angles but different widths, as shown in Fig. 2(c) and 2(d), and find that the thinner network buckles outwards while the thicker one bends inwards, indicating that the critical angle can be tuned by the arm shape.

The two distinct phenomena in Fig. 2(a) and 2(b) demonstrate a bifurcation of the active network dynamics dependent on the initial opening angles. We denote the dynamics in Fig. 2(a) and Fig. 2(b) as the buckling-dominated and merging-dominated regimes, respectively. The two regimes can be quantitatively distinguished by curvature of the network. Given a centerline profile y(x) (inset in Fig. 3), the local curvature κ is defined as $\kappa =y^{\prime \prime }/{\left(1+y^{\prime 2}\right)}^{3/2}$. We define the mean curvature [κ] along the centerline as $[\kappa ]=\int_{x_{0}}^{x_{t}}\frac{y^{\prime \prime }}{1+y^{\prime 2}}\text{d}x/\int_{x_{0}}^{x_{t}}\sqrt{1+y^{\prime 2}}\text{d}x$, where $x_{0}$ and $x_{t}$ denote the starting and ending points of the centerline, respectively. Time evolutions of mean curvatures in Fig. 2(a) and 2(b) are plotted in Fig. 3. By our definition, negative curvature represents a concave shape–characteristic of the merging-dominated regime. Additional images of concave networks are provided in Fig. S8 of the Supplemental Material. Conversely, positive curvature corresponds to a convex shape, typical of the buckling-dominated regime, where the two arms bend outward.

We conduct numerical simulations to uncover the zipping-up mechanisms. The simulations in Fig. 2 are based on a three-phase model from our previous work [8] (Supplemental Material). The simulation can reproduce the bifurcation dynamics (Fig. 2) and the simulated curvatures are in qualitative agreement with experiments (Fig. 3]). We also compute a bifurcation phase diagram by varying crosslinking rates and find that increasing the crosslinking rate promotes the network merging, thereby confirming that the merging process is driven by active crosslinking (Supplemental Material).

The boundary-layer hypothesis can explain the opening-angle dependency of the bifurcation. As θ decreases, the overlapped region expands, favoring merging at small angles. In this merging-dominated regime, the overlapped region becomes a “zipping front” that can propagate along and zip up the two network arms. It is well known that an elastic rod buckles when the compressive load exceeds a critical threshold. Buckling has also been reported in rectangular active networks [7]. The V-shaped network can be viewed as a joint of two rectangular segments, where such buckling may occur. We propose that the opening dynamics observed in Fig. 2(a) and 2(c) results from a buckling instability driven by the compressive active stress. Once buckling initiates, the two arms will only bend outwards to reduce the bending energy, which scales quadratically with local curvature [9], at the kink.

Based on the boundary-layer and buckling hypotheses, we propose a simple elastic-rod model to predict the critical angle in bifurcation. We treat the crosslinked network as an elastic rod, as shown in Fig. 4. The active stress, denoted by $\sigma_{a}$, generates a compressive force in each arm that is $F_{a}=\sigma_{a}wd$, with w and d the arm width and depth, respectively. Within the overlapped boundary layer, active crosslinking induces an attractive force $F_{h}$ between the two arms. Assuming a surface force density σ (inset in Fig. 4), the attractive force on each arm is given by $F_{h}=\sigma dh/\sin\theta$, where h is the boundary layer width. The tangential component of $F_{h}$ along each arm introduces a tension and the minimum compression within each arm is $F_{c}\approx \sigma_{a}wd-\frac{\sigma dh}{2\sin(\theta /2)}$. The bifurcation behavior of the network arises from competition between two mechanisms: buckling, characterized by $F_{c}$, and merging, characterized by $F_{h}$. We estimate the critical buckling load using the classical Euler’s result, $F_{b}=\pi^{2}EI/l^{2}$ [10], where E is the Young’s modulus, and $I=dw^{3}/12$ is the moment of inertia. The critical angle $\theta^{*}$ is determined by $F_{c}=F_{b}$, which is

(1) $$\theta^{*}=2\arcsin\frac{\sigma h}{2w\left(\sigma_{a}-CE\delta^{2}\right)},$$

where δ=w/l is the aspect ratio of each arm and $C=\pi^{2}/12$ is a constant. The network will merge when $\theta <\theta^{*}$ and buckle when $\theta >\theta^{*}$. From (1), it follows that $\theta^{*}$ can be tuned by two dimensionless geometric parameters: the ratio of boundary layer width to arm width h/w, and the aspect ratio of the arm δ=w/l. Both w and δ are programmable through light signals in experiments and simulations. When δ is fixed, increasing w will decrease the critical angle $\theta^{*}$. This is because the healing force $F_{h}$ depends only on θ and is independent of w and δ, whereas the compression $F_{c}$ in each arm scales linearly with w. As the arm width increases, a smaller $\theta^{*}$ is required to produce a greater overlapped region, and consequently a larger $F_{h}$, to balance the increasing compression. Conversely, fixing w and decreasing the aspect ratio δ also reduces $\theta^{*}$. In this case, $F_{c}$ remains unaffected by δ, but a smaller δ will make the network more slender and prone to buckling. Therefore, a smaller $\theta^{*}$ is needed to generate sufficient attractive force $F_{h}$ to suppress the elastic instability. Finally, we note that there is always overlapping of boundary layers in the tip region, rendering it always concave. The convexity and concavity predicted by our simple model (1) apply only to the bulk region away from the tip.

To test how network geometry can tune the bifurcation dynamics, we perform simulations with varying opening angles θ, arm width w, and aspect ratio δ. The outcomes—whether the network buckles or merges—are documented in the bifurcation diagrams in Fig. 5. We first fix δ=0.1 vary the arm width w in Fig. 5(a). As w increases, the critical angle $\theta^{*}$ decreases. This confirms our theory (1) that increasing network size while preserving shape makes buckling easier and merging more difficult. This is because the healing force $F_{h}$ does not scale with the network size, whereas the active compression $F_{c}$ increases linearly with w. Consequently, as the network size grows, characterized by increasing w when δ is fixed, the merging effect becomes less dominant. Similarly, Fig. 5(b) shows the bifurcation phase diagram for varying δ at fixed w, which is again consistent with our theory: as the aspect ratio δ increases, the network arms become shorter and more resistant to buckling. Both simulated phase diagrams are in quantitative agreement with experimental results. In general, h, σ, $\sigma_{a}$ and E in our theory (1) depend on local microtubule and motor concentrations. We treat them as constants in plotting the theory in Fig. 5 for simplicity. Even so, the elastic-rod model can qualitatively predict the bifurcation phase diagram and offers a clear explanation for its dependence on w and δ. Furthermore, we can define two dimensionless groups, $A=2w\sigma_{a}/\sigma h$ and $B=2CE\delta^{2}w/\sigma h$, and rewrite (1) as $\theta^{*}=2\arcsin\left[{\left(A-B\right)}^{-1}\right]$, where A is the ratio of active compression to merging force, and B is the ratio of critical buckling load to merging force. Increasing A or decreasing B makes the network easier to buckle, thereby reducing $\theta^{*}$. As an anonymous reviewer pointed out, the quantitative discrepancy between our simple model and the linear dependence of $\theta^{*}$ on δ predicted by simulations in Fig. 5b may also arise from the omission of effective surface tension in (1). This surface tension, arising from the motor activity, may cause the active network to bend inwards to reduce surface area [11]–conceptually analogous to the barreling instability described in Ref. [12].

In summary, we show that overlapping of motor boundary layers can merge defects in active networks. For V-shaped cracks, the active crosslinking also needs to overcome an elastic instability which will open up the crack, leading to a bifurcation of merging and buckling that can be tuned by the initial network geometry. It has been increasingly evident that cytoskeletal networks are gel-like materials [13] and vulnerable to a plethora of mechanical instabilities driven by self-generated active forces. Another example is the bifurcation of in-plane bending and out-of-plane buckling instabilities found in extensile active sheets [14]. However, instabilities are not always detrimental. Cells can regulate and exploit mechanical instabilities to form functional structures, such as mitotic spindles which are shaped by a barreling-type instability [12]. Further work is needed to complete a mechanical-instability phase diagram of active networks and to uncover the regulatory mechanisms used by cells to control such instabilities.

We are grateful to Zhen-Gang Wang and Howard A. Stone for fruitful discussions and to Inna Strazhnik for illustration. This project is funded by NIH 1R35 GM118043 (MIRA), Packard Foundation, Moore Foundation, and Donna and Benjamin M. Rosen Bioengineering Center. FY also acknowledges support from BBE Divisional Postdoctoral Fellowship at Caltech.

## Supplementary Material

Supplement 1

## Figures and Tables

**FIG. 1.**
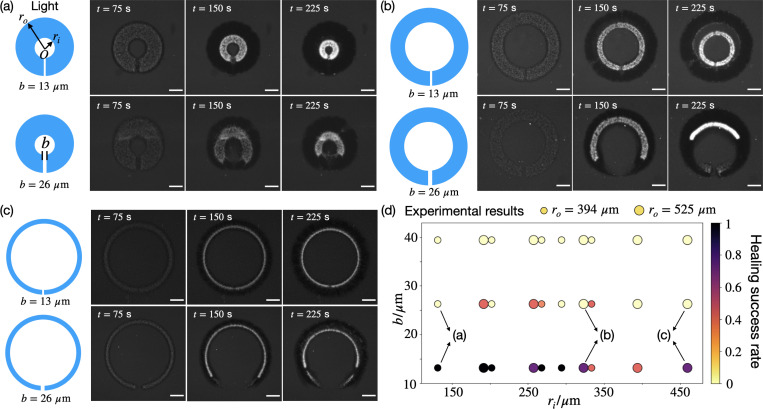
Self-healing behavior of O-shaped active networks is governed by a critical gap width. (a-c) Experimental images of O-shaped networks with gap defects. In each panel, the inner and outer radii are fixed, while two initial gap widths, b=13 *μ*m and 26 *μ*m, are shown. Scale bars, 200 *μ*m. (d) Experimental measurements of healing success rates with varying gap widths b and inner radii $r_{i}$. Small and large markers correspond to outer radii $r_{o}=394$ *μ*m and 525 *μ*m, respectively. Experiments (a-c) are labeled on the diagram.

**FIG. 2.**
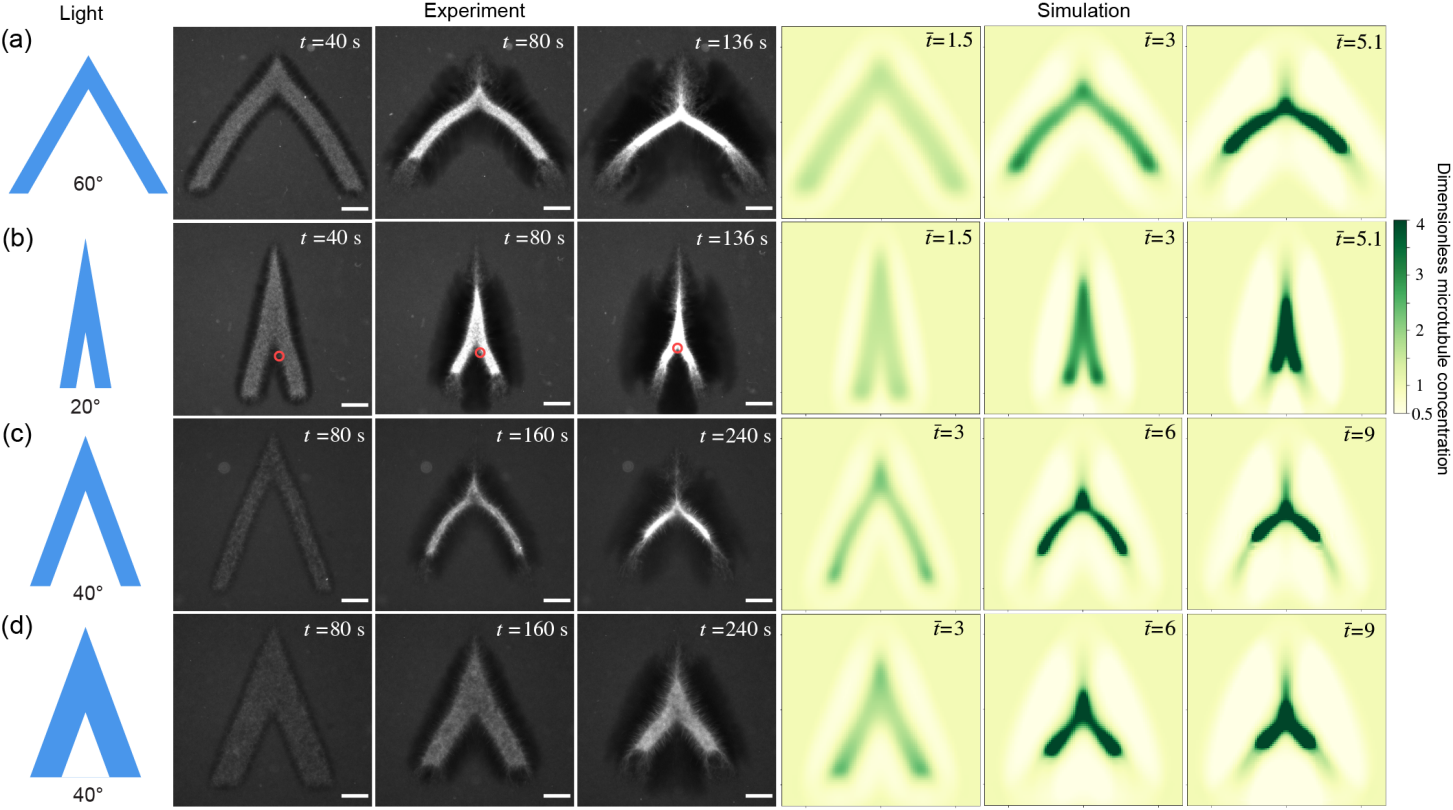
Experiments and simulations of V-shaped active networks show a bifurcation of merging- and buckling-dominated dynamics dependent on the network geometry. (a) and (b) are two networks with the same initial arm lengths and widths but different opening angles. The networks buckle at the large opening angle (a) while merge at the small angle (b). Red circles in (b) track a small protrusion on the right arm which eventually merges with the left arm, demonstrating the partial closure of the crack. (c) and (d) are two networks of the same arm lengths and opening angles but different arm widths. The thinner network (c) buckles outwards while the thicker one (d) bends inwards. The spatiotemporal dimensions of the simulated and experimental images are matched. Simulation details are in the Supplemental Material. t ($\bar{t}$) is (dimensionless) time after the first light pulse. In simulations, the microtubule concentration is non-dimensionalized by the initial microtubule concentration. Scale bar, 100 *μ*m.

**FIG. 3.**
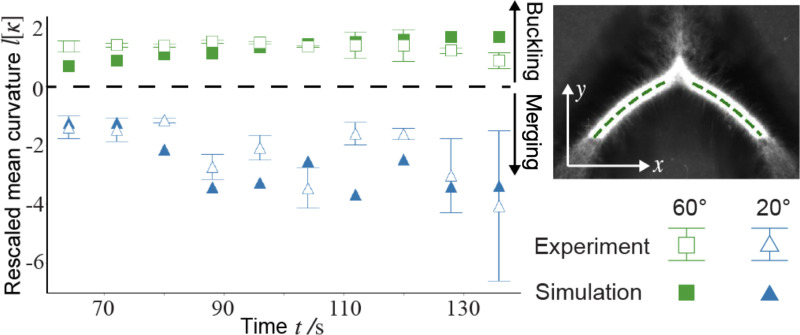
Merging and buckling-dominated dynamics can be differentiated by the network curvature. The curvature is negative (concave) for the former and positive (convex) for the latter. The mean curvature [κ] is averaged over the centerline of each arm excluding the tip region (dotted lines in the upper right inset), and rescaled by the initial arm length l. Error bars represent the difference between left and right arms in a single experiment.

**FIG. 4.**
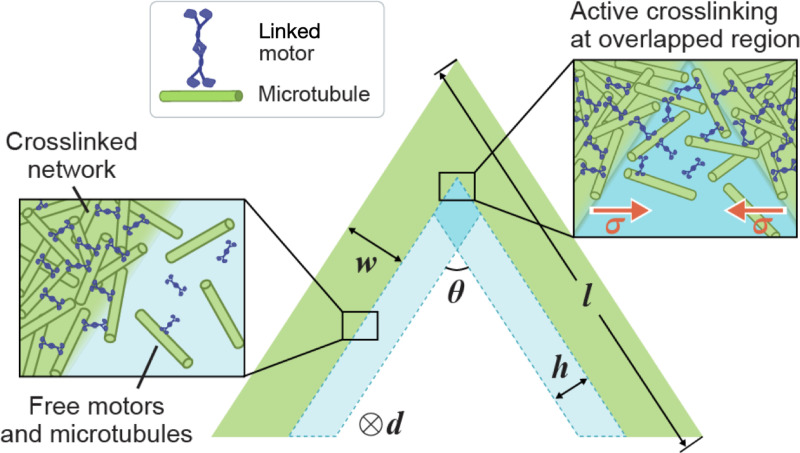
Schematic of the elastic-rod model. The crosslinked network is modeled as a kinked elastic rod (green). There are boundary layers (light blue) of free motors and microtubules next to the network surfaces. Active crosslinking takes place at the overlapped region (dark blue) of the two boundary layers.

**FIG. 5.**
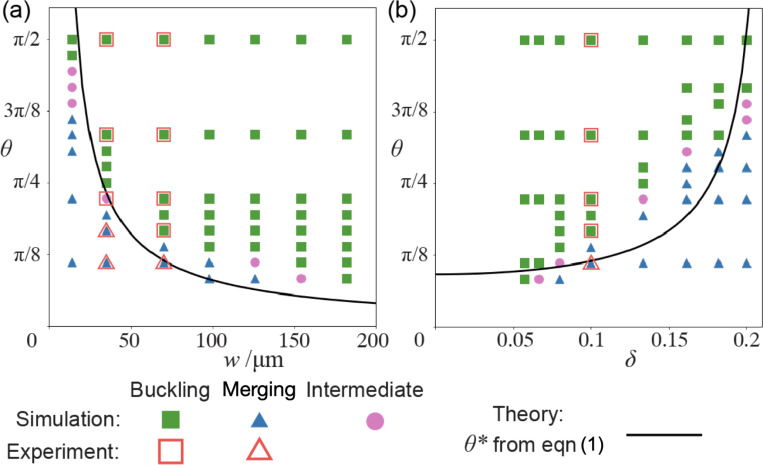
Bifurcation phase diagrams can be qualitatively predicted by the elastic-rod model. We fix δ=0.1 in (a) and w=70 *μ*m in (b). The fitting parameters used to plot the theoretical curves are $\sigma h/\sigma_{a}=20$ *μ*m and σh/CE=1 *μ*m. “Intermediate” represents when the network does not buckle and also does not show significant merging, such as Fig. 2(d). In simulations, the “Intermediate” state is characterized by the average curvature close to 0.
