# Supplementary material for: Active Healing of Microtubule-Motor Networks
Source: ArXiv. 2025 May 22:arXiv:2407.00842v2. Preprint. [Version 2] (PMC12136475)
Supplement: Supplementary file 1 [file NIHPP2407.00842v2-supplement-1.pdf]

# Supplemental Material

## CONTINUUM MODEL

In this section we derive a continuum model for the coupled dynamics of active matter and solvent flow. The model details are first published in our previous work [8], where a brief introduction and history of field-theory models on active networks are also included. We reformulate the model below in a more pedagogical manner for readers' convenience.

### Overview

The main chemical components in our system are microtubules, motors and ATPs. The dynamics of active matter and solvent flows is initiated by a cascade of chemical reactions. Firstly, the motors are activated by light and reversibly linked. The linked motors then crosslink microtubules into a contracting network, which further drives a solvent flow. In our modeling, microtubules and motors are classified according to their chemical structures, such as unlinked or linked motors, and also to their crosslinking status, such as bound linked motors and free linked motors, as well as crosslinked and free microtubules. Here ”bound” means the motors are attached to the crosslinked microtubules, and ”free” means the microtubules or motors are not attached to the crosslinked network.

All the chemical reactions mentioned above, namely the reversible “linking” of motors and the crosslinking of microtubules can be modeled in continuity equations of these chemical components. The light patterns enter our model through the dimerization rate $p^d(x, y, t)$, where $x$ and $y$ are the spatial coordinates and $t$ is time. The shape of the light region can thereby be determined by a spatiotemporal function $p^d(x, y, t)$.

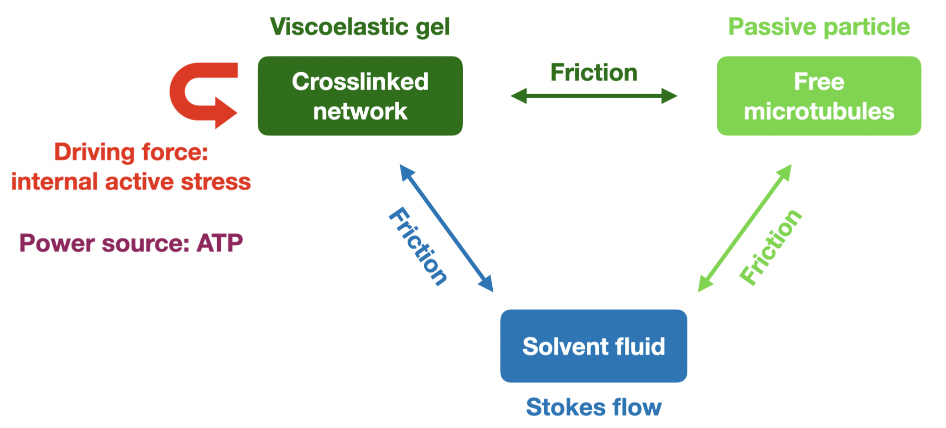


FIG. S1. **Overview of the three-phase continuum model.**

Momentum equations determine the motion of active matter and solvent from their stresses and material properties. In principle, we can write down momentum equations for each chemical species. However, small proteins such as free motors have little mechanical impact on the dynamics and can be assumed to follow the solvent flow. The crosslinked microtubules and bound linked motors can be modeled together as a single “phase”, which is termed the “crosslinked network”. The crosslinked network is modeled as a viscoelastic gel, which self-contracts driven by its internal active stresses; the solvent flow is modeled as a Stokes flow driven by the contraction of the active gel and balanced by the hydrodynamic resistance in the flow cell; the free microtubules are modeled as passive particles. Each of the three phases exert friction on the other two phases. A summary of the three phases is shown in Fig. S1.

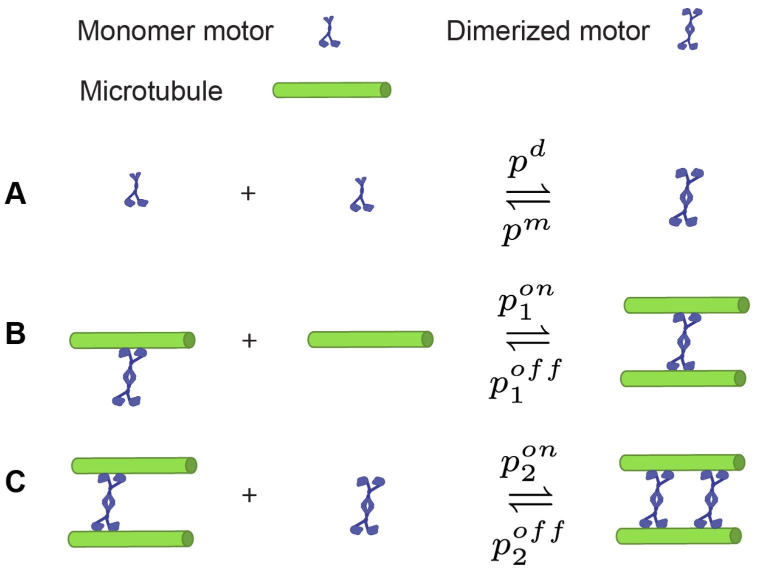


FIG. S2. **Illustrations of chemical reactions in the model**. **A**, Reversible linking of motors. **B**, Reversible crosslinking of a freely-moving microtubule $i$ and another microtubule $j$. The latter can be either free or pre-crosslinked. **C**, Reversible binding of a linked motor on two pre-crosslinked microtubules.

### Continuity equations

The continuity equations track the change of mass of a component in time due to fluxes and chemical reactions. A general form of continuity equations can be written as

$$\frac{\partial c^*}{\partial t} + \boldsymbol{\nabla} \cdot \boldsymbol{j}^* = \{\text{chemical-reaction terms}\}, \tag{S1}$$

where the asterisk (*) is used to represent the general form of any chemical species in our system, $t$ is time, $c^*$ is concentration, and $\boldsymbol{j}^*$ is flux. In our system, the flux $\boldsymbol{j}^*$ usually consist of two components, i.e.,

$$\boldsymbol{j}^* = c^* \boldsymbol{v}^* - D^* \boldsymbol{\nabla} c^*, \tag{S2}$$

where $\boldsymbol{v}^*$ is velocity and $D^*$ is diffusivity. $c^*\boldsymbol{v}^*$ and $-D^*\boldsymbol{\nabla}c^*$ are called the convective and diffusive fluxes, respectively. We follow a convention to keep the convective flux on the left-hand side of (S1) while moving the diffusive flux to the right-hand side. Therefore, the general form of continuity equations is

$$\frac{\partial c^*}{\partial t} + \boldsymbol{\nabla} \cdot (c^* \boldsymbol{v}^*) = \{\text{chemical-reaction terms}\} + D^* \nabla^2 c^* \tag{S3}$$

The chemical reactions in our system are sketched in Fig. S2. The motor proteins can reversibly link and the continuity equation is

$$\frac{\partial m}{\partial t} + \boldsymbol{\nabla} \cdot (m\boldsymbol{u}) = 2(p^m d_f - p^d m^2) + D_m \nabla^2 m \tag{S4}$$

where $m$ is the unlinked motor concentration, $d_f$ is the free linked motor concentration, $\boldsymbol{u}$ is the solvent flow velocity, $D_m$ is the diffusivity, $p^m$ and $p^d(x, y, t)$ are the monomerization and dimerization rates, respectively. The function $p^d(x, y, t)$ is determined by the light pattern. In experiments, we use V-shaped light patterns, which can be defined by a set of points $A_l = \{(x, y) \in \text{light pattern}\}$. The light pattern is projected periodically onto the flow cell with time interval $T$ and duration $\Delta t$. Naively, the dimerization rate can be written as

$$p_i^d(x, y, t) = \begin{cases} p_0^d & (x, y) \in A_l \text{ and } t \in [nT, nT + \Delta t], \\ 0 & \text{otherwise}, \end{cases} \tag{S5}$$

where $p_0^d$ is a constant dimerization rate and $n = 0, 1, 2, \ldots$ is the number of light pulses. However, directly using (S5) in simulations will cause numerical instability due to discontinuity of $p_i^d(x, y, t)$ in space. There are multiple ways to deal with this. In this Letter, we use a Gaussian kernel smoother to make (S5) a smooth function in space. The Gaussian kernel is defined as

$$K_r(x, y, X, Y) = e^{-\frac{(x-X)^2+(y-Y)^2}{2r^2}} \tag{S6}$$

with a constant $r$, which is the "kernel radius". Denoting the whole calculation domain as $A$, the smooth dimerization rate $p^d(x, y, t)$ can be obtained from applying the smoother (S6) on (S5) through

$$p^d(x, y, t) = \frac{\int_A p_i^d(X, Y, t) K_r(x, y, X, Y) \mathrm{d}X \mathrm{d}Y}{\int_A K_r(x, y, X, Y) \mathrm{d}X \mathrm{d}Y}. \tag{S7}$$

The slope of $p^d(x, y, t)$ at the boundary of light pattern $A_l$ can be tuned by the parameter $r$. A smaller $r$ yields a larger slope and thereby a sharper light boundary in simulations.

The continuity equations for the crosslinked ($c$) and free ($c_f$) microtubules are

$$\frac{\partial c}{\partial t} + \boldsymbol{\nabla} \cdot (c\boldsymbol{v}) = p_1^{\mathrm{on}} c_f d_f - p_1^{\mathrm{off}} c, \tag{S8a}$$

$$\frac{\partial c_f}{\partial t} + \boldsymbol{\nabla} \cdot (c_f \boldsymbol{v}_f) = -p_1^{\mathrm{on}} c_f d_f + p_1^{\mathrm{off}} c + D_f \nabla^2 c_f. \tag{S8b}$$

where $D_f$ is the diffusivity of the free microtubules, $p_1^{\mathrm{on}}$ and $p_1^{\mathrm{on}}$ are the crosslinking and un-crosslinking rates, respectively, see Fig. S2B. The velocities of the crosslinked and free microtubules are denoted by $\boldsymbol{v}$ and $\boldsymbol{v}_f$, respectively. In (S8a) we neglect the diffusion flux of the crosslinked microtubules. Also Note that in writing down the chemical reaction terms in (S8), we assume the reaction orders are 1 for simplicity.

The continuity equations for the free ($d_f$) and the bound ($d_b$) linked motors are

$$\frac{\partial d_f}{\partial t} + \boldsymbol{\nabla} \cdot (d_f \boldsymbol{u}) = -p^m d_f + p^d m^2 - p_1^{\mathrm{on}} c_f d_f - p_2^{\mathrm{on}} c d_f + p_2^{\mathrm{off}} d_b + D_d \nabla^2 d_f \tag{S9a}$$

$$\frac{\partial d_b}{\partial t} + \boldsymbol{\nabla} \cdot (d_b \boldsymbol{v}) = p_1^{\mathrm{on}} c_f d_f + p_2^{\mathrm{on}} c d_f - p_2^{\mathrm{off}} d_b, \tag{S9b}$$

where $d_b$ is the bound linked motor concentration, $p_2^{\mathrm{on}}$ is the rate of the linked motors binding on the crosslinked microtubules, $D_d$ is the diffusivity of the free linked motors, $p_2^{\mathrm{off}}$ is the unbinding rate of a linked motor that does not uncrosslink the two microtubules, see Fig. S2C. All free motors are assumed to follow the solvent flow ($\boldsymbol{u}$) and the bound motors follow the crosslinked gel ($\boldsymbol{v}$) .

The continuity equation for the ATP is

$$\frac{\partial c_A}{\partial t} + \boldsymbol{\nabla} \cdot (c_A \boldsymbol{u}) = -k_A d_b c_A + D_A \nabla^2 c_A, \tag{S10}$$

where $k_A$ is the consumption rate of the ATP by the bound linked motors, and $D_A$ is the ATP diffusivity.

The solvent flow is incompressible, meaning that its density does not change. The continuity equation for the solvent flow is just

$$\boldsymbol{\nabla} \cdot \boldsymbol{u} = 0. \tag{S11}$$

**Momentum equations**

Momentum equations are the balance between the momentum fluxes, stresses and body forces in a differential form. At low Reynolds number, inertial momentum fluxes are usually neglected. Under this assumption, a general form of the momentum equation is

$$\boldsymbol{\nabla} \cdot \boldsymbol{\sigma}^* + \boldsymbol{f}^* = \boldsymbol{0}, \tag{S12}$$

where $\boldsymbol{\sigma}^*$ is the total stress and $\boldsymbol{f}^*$ is the total body force. In this section, we list the momentum equations for each phase.

*Crosslinked network as an active gel*

The crosslinked network forms an active gel, which is assumed to be viscoelastic. The momentum equation can be written as

$$\boldsymbol{\nabla}\cdot(\boldsymbol{\sigma}_a+\boldsymbol{\sigma}_v+\boldsymbol{\sigma}_{el}+\boldsymbol{\sigma}_{st})+\boldsymbol{f}_{fl}+\boldsymbol{f}_f=\boldsymbol{0}, \tag{S13}$$

where $\boldsymbol{\sigma}_a$, $\boldsymbol{\sigma}_v$, $\boldsymbol{\sigma}_{el}$ and $\boldsymbol{\sigma}_{st}$ are the active, viscous, elastic and steric stresses in the gel, $\boldsymbol{f}_{fl}$ and $\boldsymbol{f}_f$ are the friction with the solvent and freely-moving microtubules, respectively. Their explicit expressions can be derived from microscopic interactions [18, 19]. The active contractile stress is assumed to be

$$\boldsymbol{\sigma}_a=\alpha c_A d_b c\boldsymbol{I}, \tag{S14}$$

where $c$, $c_A$ and $d_b$ are the concentrations of the crosslinked microtubules, ATP and the bound linked motors, respectively, $\alpha$ is a constant activity coefficient and $\boldsymbol{I}$ is the identity tensor. This active stress is derived to be $\boldsymbol{\sigma}_a'\propto c^2\boldsymbol{I}$, in Ref [19], where the bound motor concentration is implicitly assumed to be uniform and does not appear in active stresses. In our experiments, the concentration difference of the linked motors inside and outside of the illuminated regions is the key to form active gels. We can modify the theory in Ref [19] by adding a prefactor $d_b/c$ in the active stresses, which is the number of bound motors per microtubule.

The viscous stress is

$$\boldsymbol{\sigma}_v=\eta d_b c\left(\boldsymbol{\nabla v}+\boldsymbol{\nabla v}^T\right), \tag{S15}$$

where $\boldsymbol{v}$ is the velocity of the crosslinked microtubules and $\eta$ is the viscosity coefficient. This viscous stress in Ref [19] is $\boldsymbol{\sigma}_v'\propto c^2\left(\boldsymbol{\nabla v}+\boldsymbol{\nabla v}^T\right)$. In our model the coefficient is modified from $c^2$ to $d_b c$ to incorporate the effects of the motor concentrations.

The elastic stress $\boldsymbol{\sigma}_{el}$ is assumed to follow the Oldroyd-Maxwell model with a long relaxation time [20],

$$\frac{\mathrm{D}\boldsymbol{\sigma}_{el}}{\mathrm{D}t}-\left[\boldsymbol{\sigma}_{el}\cdot\boldsymbol{\nabla v}+\boldsymbol{\nabla v}^T\cdot\boldsymbol{\sigma}_{el}\right]=\eta_{el}d_b c\left(\boldsymbol{\nabla v}+\boldsymbol{\nabla v}^T\right)-p_1^{\mathrm{off}}\boldsymbol{\sigma}_{el}, \tag{S16}$$

where $\eta_{el}$ is the elasticity coefficient and $\mathrm{D}/\mathrm{D}t$ is the material derivative. We choose the Oldroyd-Maxwell model for its simplicity.

To avoid unlimited density build-up at one point, we add a steric stress that is

$$\boldsymbol{\sigma}_{st}=-\xi c^2\boldsymbol{I}, \tag{S17}$$

with a constant coefficient $\xi$.

The friction with freely-moving microtubules is

$$\boldsymbol{f}_f=\beta c c_f(\boldsymbol{v}_f-\boldsymbol{v}), \tag{S18}$$

where $\beta$ is the friction coefficient. The friction with the solvent flow is

$$\boldsymbol{f}_{fl}=\gamma c(\boldsymbol{u}-\boldsymbol{v}) \tag{S19}$$

with the drag coefficient $\gamma$. In general the hydrodynamic drag coefficient depends on the fiber orientation [21]. In this Letter we neglect the polarity in both active stresses and hydrodynamic friction.

*Free microtubules*

The free microtubules are passive particles that only experience friction from the gel and the ambient fluid. The force balance is $\beta c_f c\,(\boldsymbol{v}-\boldsymbol{v}_f)+\gamma c_f(\boldsymbol{u}-\boldsymbol{v}_f)=\boldsymbol{0}$, and their velocity is therefore

$$\boldsymbol{v}_f=\frac{\beta c\boldsymbol{v}+\gamma\boldsymbol{u}}{\beta c+\gamma}. \tag{S20}$$

*Solvent flows*

The flow cells used in our experiments are usually called Hele-Shaw cells in fluid mechanics, meaning that their horizontal dimensions ($x-$ and $y-$direction) greatly exceed their vertical dimension ($z$-direction). We now derive the averaged two-dimensional flow equation in the $xy-$plane. We start from the three-dimensional Stokes equation, that is,

$$-\widetilde{\boldsymbol{\nabla}}\widetilde{\Pi} + \mu\widetilde{\nabla}^2\widetilde{\boldsymbol{u}} + \gamma\widetilde{c}\left(\widetilde{\boldsymbol{v}} - \widetilde{\boldsymbol{u}}\right) + \gamma\widetilde{c}_f\left(\widetilde{\boldsymbol{v}}_f - \widetilde{\boldsymbol{u}}\right) = 0, \tag{S21}$$

where $\widetilde{\Pi}$ and $\mu$ are the fluid pressure and viscosity, respectively. We use a tilde "$\sim$" on top to indicate the variable is a function of $(x, y, z)$ and if the variable is a vector, it is a three-dimensional vector, e.g., $\widetilde{\Pi} = \widetilde{\Pi}(x, y, z)$ and $\widetilde{\boldsymbol{u}}(x, y, z) = (\widetilde{u}_x(x, y, z), \widetilde{u}_y(x, y, z), \widetilde{u}_z(x, y, z))$. Since the vertical length scale is much smaller than horizontal length scales, the classical lubrication theory [21] shows that $\widetilde{\Pi}(x, y, z) \approx \Pi(x, y)$, i.e., the fluid pressure is constant along the $z$-direction, and $\widetilde{u}_z \approx 0$. Substituting these two results into equation (S21) yields $\widetilde{v}_z \approx 0$ and $\widetilde{v}_{f,z} \approx 0$. Furthermore, the $x-$ and $y-$components of equation (S21) can be approximated by

$$-\boldsymbol{\nabla}\Pi + \mu\frac{\partial^2\widetilde{\boldsymbol{u}}}{\partial z^2} + \gamma\widetilde{c}\left(\widetilde{\boldsymbol{v}} - \widetilde{\boldsymbol{u}}\right) + \gamma\widetilde{c}_f\left(\widetilde{\boldsymbol{v}}_f - \widetilde{\boldsymbol{u}}\right) = 0, \tag{S22}$$

where all vectors only have $x-$ and $y-$components, i.e., $\widetilde{\boldsymbol{u}} = (\widetilde{u}_x(x, y, z), \widetilde{u}_y(x, y, z))$, $\widetilde{\boldsymbol{v}} = (\widetilde{v}_x(x, y, z), \widetilde{v}_y(x, y, z))$ and $\widetilde{\boldsymbol{v}}_f = (\widetilde{v}_{f,x}(x, y, z), \widetilde{v}_{f,y}(x, y, z))$. Therefore, the analytical solution of $\widetilde{\boldsymbol{u}}$ requires knowledge of the three-dimensional distribution of $\widetilde{c}$, $\widetilde{\boldsymbol{v}}$ and $\widetilde{\boldsymbol{v}}_f$. To derive a two-dimensional model, we further simplify equation (S22) by replacing the friction with their $z$-directional average, i.e.,

$$-\boldsymbol{\nabla}\Pi + \mu\frac{\partial^2\widetilde{\boldsymbol{u}}}{\partial z^2} + \gamma c\left(\boldsymbol{v} - \boldsymbol{u}\right) + \gamma c_f\left(\boldsymbol{v}_f - \boldsymbol{u}\right) = 0, \tag{S23}$$

where $c(x, y) = h^{-1}\int_0^h \widetilde{c}\mathrm{d}z$, $\boldsymbol{v}(x, y) = h^{-1}\int_0^h \widetilde{\boldsymbol{v}}\mathrm{d}z$, $\boldsymbol{v}_f(x, y) = h^{-1}\int_0^h \widetilde{\boldsymbol{v}}_f\mathrm{d}z$, and $\boldsymbol{u}(x, y) = h^{-1}\int_0^h \widetilde{\boldsymbol{u}}\mathrm{d}z$ with the top and bottom walls of channel at $z = 0$ and $z = h$, respectively. These notations are consistent with the rest of the Letter. The solution of equation (S23) is

$$\widetilde{\boldsymbol{u}} = \frac{1}{2\mu}\left[\boldsymbol{\nabla}\Pi - \gamma c\left(\boldsymbol{v} - \boldsymbol{u}\right) - \gamma c_f\left(\boldsymbol{v}_f - \boldsymbol{u}\right)\right] z(z - h). \tag{S24}$$

Combining $\boldsymbol{u} = h^{-1}\int_0^h \widetilde{\boldsymbol{u}}\mathrm{d}z$ and equations (S20) and (S24), we have the equation for the averaged two-dimensional flow velocity

$$\boldsymbol{u} = -\frac{h^2}{12\mu}\left[\boldsymbol{\nabla}\Pi - \left(\gamma c + \gamma_f c_f\right)\left(\boldsymbol{v} - \boldsymbol{u}\right)\right], \tag{S25}$$

with $\gamma_f = \gamma\beta c/(\beta c + \gamma)$. The above result is a modified Darcy's law in a Hele-Shaw cell [21] by incorporating the microtubule friction.

**Non-dimensionalization**

Using the initial microtubule concentration $c_0$, initial unlinked motor concentration $m_0$, initial ATP concentration $c_{A0}$, and the typical length scale of the illuminated region $l$, we can non-dimensionalize the governing equations by

$$\begin{gathered}\bar{c} = \frac{c}{c_0}, \quad \overline{\boldsymbol{\nabla}} = l_0\boldsymbol{\nabla}, \quad \overline{m} = \frac{m}{m_0}, \quad \bar{d}_f = \frac{d_f}{m_0}, \quad \bar{d}_b = \frac{d_b}{m_0}, \quad \bar{c}_A = \frac{c_A}{c_{A0}} \\ \bar{t} = \frac{t}{t_0}, \quad \overline{\boldsymbol{v}} = \frac{\boldsymbol{v}}{v_0}, \quad \overline{\boldsymbol{v}}_f = \frac{\boldsymbol{v}_f}{v_0}, \quad \overline{\boldsymbol{u}} = \frac{\boldsymbol{u}}{v_0}, \quad \overline{\boldsymbol{\sigma}}_{el} = \frac{\boldsymbol{\sigma}_{el}}{\sigma_0}, \quad \overline{\Pi} = \frac{\Pi}{\Pi_0},\end{gathered} \tag{S26}$$

where we use overlines to denote dimensionless variables, $t_0$, $v_0$, $\sigma_0$ and $\Pi_0$ are typical scales of time, velocity, stress, and fluid pressure, respectively. By balancing the contractile stress (S14) and the viscous stress (S15) in the active gel, we have $v_0 = \alpha c_{A0} l/\eta$. The typical time scale follows as $t_0 = l_0/v_0 = \eta/\alpha c_{A0}$. The typical stress scale can be obtained from equation (S14), which is $\sigma_0 = \alpha c_{A0} c_0 m_0$. From equation (S25), we have $\Pi_0 = c_0\gamma v_0 l_0 = \alpha\gamma c_0 c_{A0} l_0^2/\eta$.

The dimensionless continuity equations for the unlinked (S4) and linked (S9) motors are

$$\frac{\partial \overline{m}}{\partial \overline{t}} + \overline{\boldsymbol{\nabla}} \cdot (\overline{m}\overline{\boldsymbol{u}}) = 2(\overline{p}^m \overline{d}_f - \overline{p}^d \overline{m}^2) + \mathrm{Pe}_m^{-1} \overline{\nabla}^2 \overline{m}, \tag{S27a}$$

$$\frac{\partial \overline{d}_f}{\partial \overline{t}} + \overline{\boldsymbol{\nabla}} \cdot \left(\overline{d}_f \overline{\boldsymbol{u}}\right) = -\overline{p}^m \overline{d}_f + \overline{p}^d \overline{m}^2 - \overline{p}_1^{\mathrm{on}} \overline{c}_f \overline{d}_f - \overline{p}_2^{\mathrm{on}} \overline{c} \overline{d}_f + \overline{p}_2^{\mathrm{off}} \overline{d}_b + \mathrm{Pe}_d^{-1} \overline{\nabla}^2 \overline{d}_f, \tag{S27b}$$

$$\frac{\partial \overline{d}_b}{\partial \overline{t}} + \overline{\boldsymbol{\nabla}} \cdot \left(\overline{d}_b \overline{\boldsymbol{v}}\right) = \overline{p}_1^{\mathrm{on}} \overline{c}_f \overline{d}_f + \overline{p}_2^{\mathrm{on}} \overline{c} \overline{d}_f - \overline{p}_2^{\mathrm{off}} \overline{d}_b, \tag{S27c}$$

where the dimensionless reaction coefficients are $\overline{p}^m = p^m t_0$, $\overline{p}^d = p^m m_0 t_0$, $\overline{p}_1^{\mathrm{on}} = p_1^{\mathrm{on}} c_0 t_0$, $\overline{p}_2^{\mathrm{on}} = p_2^{\mathrm{on}} c_0 t_0$ and $\overline{p}_2^{\mathrm{off}} = p_2^{\mathrm{off}} t_0$. We use Pe to denote the Péclet number and $\mathrm{Pe}_m = v_0 l / D_m$, $\mathrm{Pe}_d = v_0 l / D_d$.

The dimensionless continuity equations for the microtubules (S8) are

$$\frac{\partial \overline{c}}{\partial \overline{t}} + \overline{\boldsymbol{\nabla}} \cdot (\overline{c}\overline{\boldsymbol{v}}) = \overline{p}_1^{\mathrm{on}} \overline{c}_f \overline{d}_f - \overline{p}_1^{\mathrm{off}} \overline{c}, \tag{S28a}$$

$$\frac{\partial \overline{c}_f}{\partial \overline{t}} + \overline{\boldsymbol{\nabla}} \cdot (\overline{c}_f \overline{\boldsymbol{v}}_f) = -\overline{p}_1^{\mathrm{on}} \overline{c}_f \overline{d}_f + \overline{p}_1^{\mathrm{off}} \overline{c} + \mathrm{Pe}_f^{-1} \overline{\nabla}^2 \overline{c}_f \tag{S28b}$$

with $\overline{p}_1^{\mathrm{off}} = p_1^{\mathrm{off}} t_0$, $\mathrm{Pe}_f = v_0 l_0 / D_f$. For the ATP, equation (S10) becomes

$$\frac{\partial \overline{c}_A}{\partial \overline{t}} + \overline{\boldsymbol{\nabla}} \cdot (\overline{c}\overline{\boldsymbol{u}}) = -\overline{k}_A \overline{d}_b \overline{c}_A + Pe_A^{-1} \overline{\nabla}^2 \overline{c}_A, \tag{S29}$$

where $\overline{k}_A = k_A m_0 t_0$ and $\mathrm{Pe}_A = v_0 l_0 / D_A$.

The continuity equation for the solvent flow (S11) is

$$\overline{\boldsymbol{\nabla}} \cdot \overline{\boldsymbol{u}} = 0. \tag{S30}$$

Using equations (S13-S20), the dimensionless momentum equations for the active gel are

$$\overline{\boldsymbol{\nabla}} \cdot \left[\left(\overline{c}_A \overline{d}_b \overline{c} - \overline{\xi} \overline{c}^2\right) \boldsymbol{I} + \overline{\boldsymbol{\nabla}} \overline{\boldsymbol{v}} + \overline{\boldsymbol{\nabla}} \overline{\boldsymbol{v}}^T + \overline{\boldsymbol{\sigma}}_{el}\right] + \left(\overline{\gamma}_f \overline{c}_f + \overline{\gamma} \overline{c}\right) (\overline{\boldsymbol{u}} - \overline{\boldsymbol{v}}) = \boldsymbol{0} \tag{S31}$$

where $\overline{\xi} = \xi c_0 / \alpha c_{A0} m_0$, $\overline{\gamma} = \gamma l_0^2 / m_0 \eta$, $\overline{\gamma}_f = \gamma_f l_0^2 / m_0 \eta$ and

$$\frac{\mathrm{D}\overline{\boldsymbol{\sigma}}_{el}}{\mathrm{D}\overline{t}} - \left[\overline{\boldsymbol{\sigma}}_{el} \cdot \overline{\boldsymbol{\nabla}} \overline{\boldsymbol{v}} + \overline{\boldsymbol{\nabla}} \overline{\boldsymbol{v}}^T \cdot \overline{\boldsymbol{\sigma}}_{el}\right] = \overline{\eta}_{el} \overline{d}_b \overline{c} \left(\overline{\boldsymbol{\nabla}} \overline{\boldsymbol{v}} + \overline{\boldsymbol{\nabla}} \overline{\boldsymbol{v}}^T\right) - \overline{p}_1^{\mathrm{off}} \overline{\boldsymbol{\sigma}}_{el}, \tag{S32}$$

with $\overline{\eta}_{el} = \eta_{el} / \alpha c_{A0}$.

The dimensionless velocity of the freely-moving microtubules (S20) is

$$\overline{\boldsymbol{v}}_f = \frac{\overline{\beta} \overline{c} \overline{\boldsymbol{v}} + \overline{\gamma} \overline{\boldsymbol{u}}}{\overline{\beta} \overline{c} + \overline{\gamma}}, \tag{S33}$$

with $\overline{\beta} = \beta l_0^2 c_0 / m_0 \eta$. Note that only two of $\overline{\beta}$, $\overline{\gamma}$ and $\overline{\gamma}_f$ are independent, which are connected through $\overline{\gamma}_f = \overline{\gamma} \overline{\beta} \overline{c} / (\overline{\beta} \overline{c} + \overline{\gamma})$.

The dimensionless solvent flow (S25) is

$$\overline{\boldsymbol{u}} = -\overline{\zeta} \left[\overline{\boldsymbol{\nabla}} \Pi - \left(\overline{c} + \frac{\overline{\gamma}_f}{\overline{\gamma}} \overline{c}_f\right) (\overline{\boldsymbol{v}} - \overline{\boldsymbol{u}})\right], \tag{S34}$$

with $\overline{\zeta} = h^2 c_0 \gamma / 12\mu$.

## Simulations

We used the finite difference method in numerical simulations with the central difference scheme in space and the method of lines in time. The codes are written in Python and available at https://github.com/fy26/ActiveHealing.

The parameters used in simulations are

$$\begin{gathered} l_0 = 220\ \mu\mathrm{m}, \quad t_0 = 40\ \mathrm{s}, \quad p_0^d = 15, \quad p^m = 12, \quad p_1^{\mathrm{on}} = 15, \quad p_2^{\mathrm{on}} = 0.75, \\ p_1^{\mathrm{off}} = 10, \quad p_2^{\mathrm{off}} = 20, \quad \mathrm{Pe}_f^{-1} = 10^{-3}, \quad \mathrm{Pe}_m^{-1} = \mathrm{Pe}_d^{-1} = \mathrm{Pe}_A^{-1} = 10^{-2}, \\ \overline{k}_A = 0.8, \quad \overline{\xi} = 0.08, \quad \overline{\eta}_{el} = 1.5, \quad \overline{\gamma} = 0.045, \quad \overline{\beta} = 0.45, \quad \overline{\zeta} = 20.5. \end{gathered} \tag{S35}$$

In addition, the duration of each light pulse, $\Delta t / t_0 = 0.04$ and the time interval between two light pulses is $T / t_0 = 0.2$.

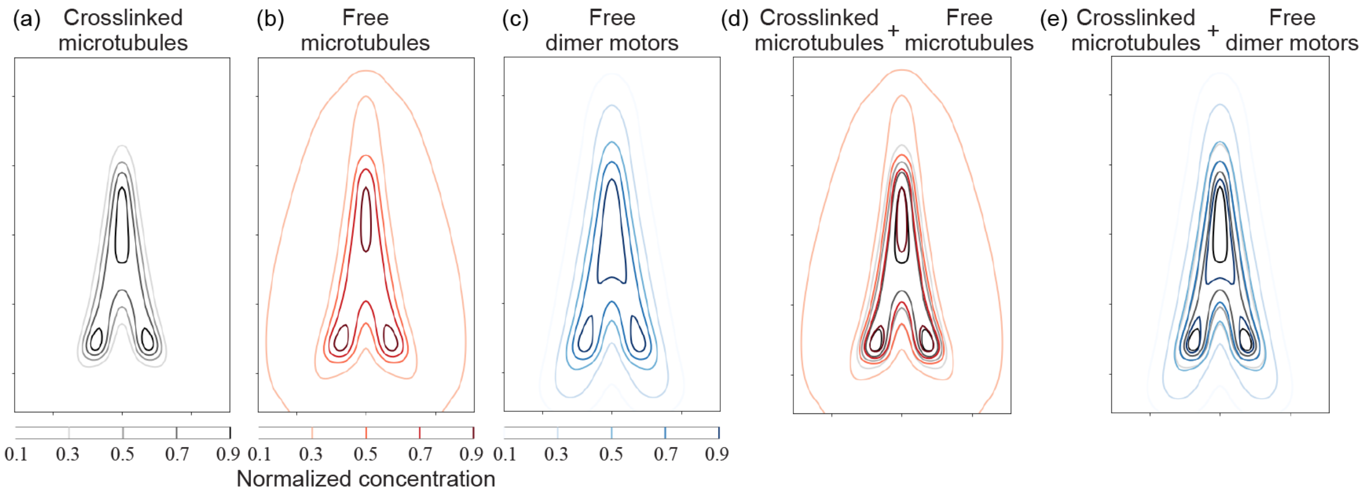


FIG. S3. Normalized concentration contours of crosslinked microtubules, free microtubules and free linked motors. The contours of crosslinked microtubules (a) are encapsulated by the contours of of free microtubules (b) and free linked motors (c) at the same levels, demonstrating that there is a boundary layer of free microtubules and motors outside the network surface. (e) and (f) are overlaid contours of crosslinked and free microtubules, and crosslinked and free linked motors, respectively.

*Boundary layer of free microtubules and motors*

We plot normalized concentrations of crosslinked microtubules, free microtubules and free linked motors in Fig. S3. The contours are simulated results of Fig 2(b) in the main text at $\bar{t} = 3$. From Fig. S3 we can see that the contours of crosslinked microtubules are always encapsulated by the contours of free microtubules and motors, confirming that there is a boundary layer at the surface of active networks.

**Force balance in the elastic-rod model.**

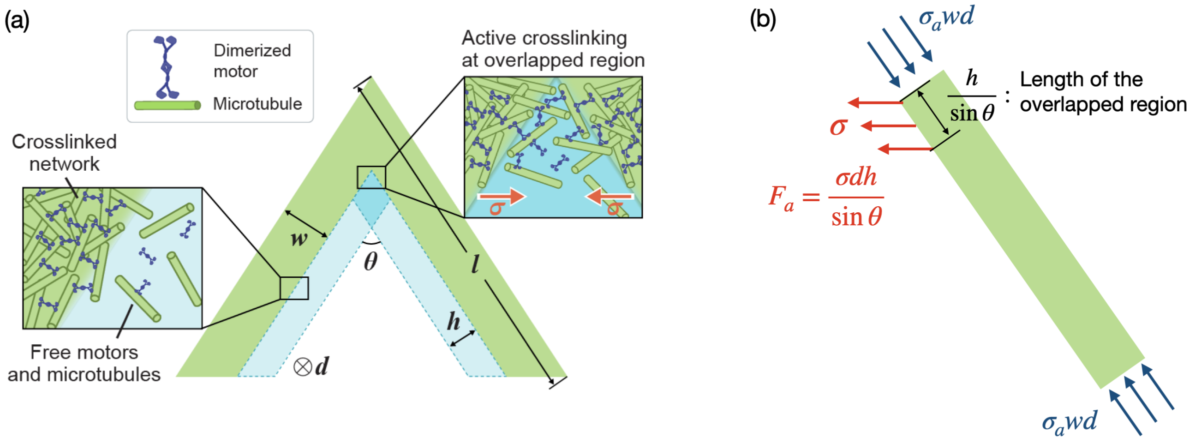


FIG. S4. (a) Schematics of the elastic-rod model, identical to Fig. 5 in the main text. (b) Schematics of the active healing force $F_a = \sigma dh/\sin\theta$ and the active compression $\sigma_a wd$ on the right arm. The viscous force exerted by the background solution on the arm is not plotted.

To illustrate how forces act on each arm in the elastic-rod model, we depict the active healing force $F_h = \sigma dh/\sin\theta$ and the active compression $\sigma_a wd$ in Fig. S4.

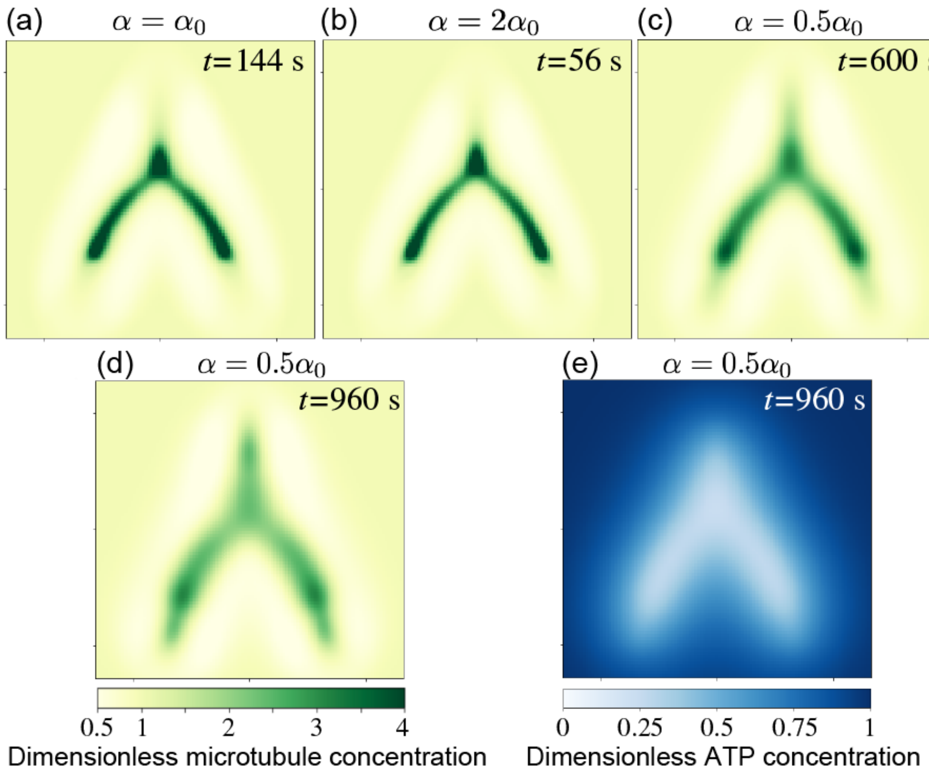


FIG. S5. Simulations with different activities $\alpha$ show that the activity does not affect the arm curvatures but can change the time scales and densities of the active networks. Simulation in (a) is the same as Fig. 2(c) in the main text and used as a benchmark. Increasing (b) or decreasing (c) the activity $\alpha$ can significantly speed up or slow down the active matter dynamics, respectively. (d) At late times, smaller activity yields a loosely crosslinked network. (e) Depletion of ATP corresponding to (d). The ATP concentration is non-dimensionalized by the initial ATP concentration.

## Motor activity and active healing

How does activity influence the healing process? The active stress $\boldsymbol{\sigma}_a$ in our simulations is modeled as an isotropic contractile stress $\sigma_a = \alpha c_A d_b c \boldsymbol{I}$, where $\alpha$ is the activity, $c_A$ is the ATP concentration, and $d_b$ is the concentration of motors bound to the active network. We numerically study how the activity $\alpha$ affects the bifurcation dynamics. Using the simulation in Fig. 2(c) in the main text as a benchmark, we find that decreasing activity can slow down the active matter dynamics (Fig. S5). The smaller activity also yields a more loosely crosslinked network at late times, as shown in Fig. S5(d), in contrast with the dense networks in Fig. 2(c) in the main text. However, the activity does not affect the arm curvatures, as seen from Fig. S5(a-c). We also vary the activity on networks with different opening angles and get the same conclusions. The unvarying curvatures indicate that changing activity does not impact the competition of the merging and buckling forces and therefore does not change the critical angle $\theta^*$ in bifurcation. This is not surprising as both the merging and compression forces originate from the same microscopic active stress $\boldsymbol{\sigma}_a$. Our simulations suggest that increasing activity can enhance both healing and compression forces in a similar manner such that even though the overall dynamics is accelerated, the critical angle $\theta^*$, which depends on the ratio of the compression and merging forces, remains largely unaffected. The loosely crosslinked network in Fig. S5(d) is a natural consequence of weaker contractile stresses. Additionally, the energy consumption, or equivalently, the ATP consumption also plays an important role in determining the late-stage network densities. In our simulations, the ATP consumption rate is modeled as $-k_A d_b c_A$ with a constant coefficient $k_A$ (see equation (S10)). Note that we assume $k_A$ is unrelated to the activity $\alpha$. Since a smaller activity prolongs the dynamics and consumes more ATP over a longer time span, at late times the depletion of ATP, as shown in Fig. S5(e), will stall further contraction of the network, which also gives rise to a more loosely crosslinked network.

## EXPERIMENTAL SETUP

### Light-induced kinesin expression and purification

We constructed two chimeras of D. melanogaster kinesin K401: K401-iLID and K401-micro as previously described (Addgene 122484 and 122485) [7]. In short, for the K401-iLiD plasmid we inserted iLID with a His tag after the C-terminus of K401. For the K401-micro plasmid, we inserted K401 between the His-MBP and the micro. The MBP domain is needed to ensure the microdomain remains fully functional during expression [22]. After the expression, the MBP domain can be cleaved of by TEV protease.

For protein expression, we transformed the plasmids to BL21(DE3)pLysS cells. The cells were grown in LB and induced at with 1mM IPTG at 18°C for 16 hours after reaching OD 0.6. The cells were then pelleted at 4000G and resuspended in lysis buffer (50 mM sodium phosphate, 4 mM $MgCl_2$, 250 mM NaCl, 25 mM imidazole, 0.05mM MgATP, 5 mM BME, 1 mg/ml lysozyme and 1 tablet/50 mL of Complete Protease Inhibitor). After a 1-hour incubation with stirring, the lysate was passed through a 30kPSI cell disruptor. The lysate was clarified at 30,000 G for 1 hour. The supernatant was then incubated with Ni-NTA agarose resin for 1 hour. The lysate/Ni-NTA mixture was loaded into a chromatography column and washed three times with wash buffer (Lysis buffer with no lysozyme nor complete EDTA tablet), and eluted with 500mM imidazole. Protein elutions were dialyzed overnight using 30 kDa MWCO membrane against 50 mM sodium phosphase, 4 mM $MgCl_2$, 250 mM NaCl, 0.05 mM MgATP, and 1 mM BME. For the K401-micro elution, we added TEV protease at a 1:25 mass ratio to remove the MBP domain. Then, we used centrifugal filters to exchange to pH 6.7 protein storage buffer (50 mM imidazole, HCl for pH balancing, 4 mM $MgCl_2$, 2 mM DTT, 50 $\mu$M MgATP, and 36% sucrose). Proteins were then aliquoted and flash frozen in LN2 and stored under -80°C.

### Microtubule polymerization and length distribution

Fluorscent microtubule polymerization was previously described [7]. In short, we used a protocol based on one found on the Dogic lab homepage. The procedure began by preloading and starting a 37°C water bath. GMP-cpp, reagents, and tubes were cooled on ice. A 20 mM DTT solution was prepared using Pierce no-weigh format, and a GMP mixture consisting of M2B, DTT, and GMP-cpp was made and stored on ice. The ultracentrifuge and rotor were pre-cooled to 4°C. Tubulin (20 mg/mL) and labeled tubulin (20 mg/mL) were thawed in the water bath until mostly thawed and then cooled on ice. In a cold room, labeled tubulin was added to the stock vial of unlabeled tubulin and mixed gently. The GMP mixture was then added to the tubulin mixture and stirred gently. This combined mixture was pipetted into ultracentrifuge tubes and incubated on ice for 5 minutes before being centrifuged at 90,000 rpm, 4°C for 8 minutes. The supernatant was carefully collected without disturbing the pellet and transferred to an Eppendorf tube, mixed, and stored on ice. The mixture was incubated in a 37°C water bath for 1 hour, protected from light. Aliquots were then dispensed into PCR strip tubes, which were spun to collect the fluid at the bottom. Finally, the PCR strips were flash frozen in liquid nitrogen and stored in a -80°C freezer.

To measure the length distribution of microtubules, we imaged fluorescently labeled microtubules immobilized onto the cover glass surface of a flow cell. The cover glass was treated with a 0.01% solution of poly-L-lysine (Sigma P4707) to promote microtubule binding. The lengths of microtubules were determined by image segmentation. Each microtubule image was normalized and underwent local and global thresholding to correct for non-uniform backgrounds and obtain thresholded images of putative microtubules. Morphological operations were applied to reconnect small breaks in filaments. Objects near the image boundary were removed, and small or circular objects were filtered out based on size and eccentricity thresholds. Potential microtubule crossovers were identified and eliminated by analyzing the angles of lines within the image.

### Sample chambers for experiments

Glass slides and coverslips were first cleaned using a series of washes. Slides and coverslips were placed in respective containers, and 2% Hellmanex solution was prepared by mixing 6 mL Hellmanex with 300 mL DI water, heated, and poured into the containers. The containers were sonicated for 10 minutes, followed by three DI water rinses and an ethanol rinse. Ethanol was added to the containers and sonicated again for 10 minutes, followed by another ethanol rinse and three DI water rinses. Next, 0.1 M KOH was added to the containers, sonicated for 10 minutes, and rinsed

three times with DI water. The slides were then etched overnight with 5% HCl and rinsed three times with DI water. Clean slides and coverslips were stored in DI water.

For silane coupling, a 2% acrylamide solution was prepared using 40% acrylamide stock solution, and degassed under vacuum. In a chemical hood, 98.5% ethanol, 1% acetic acid, and 0.5% silane agent were mixed to prepare the silane-coupling solution, which was immediately poured into the containers with the slides and coverslips and incubated at room temperature for 20-30 minutes. The slides were then rinsed once with ethanol, three times with DI water, and baked at 110°C for 30 minutes or 50°C overnight.

For acrylamide polymerization, the degassed 300 mL of 2% acrylamide solution was moved to a stir plate, and 105 $\mu$L TEMED and 210 mg ammonium persulfate were added. The solution was immediately poured over the silane-coupled slides and coverslips and left to polymerize overnight at 4°C. Before use, the slides and coverslips were rinsed with DI water and air-dried.

### Reaction mixture

For the self-organization experiments, K401-micro, K401-iLID, and microtubules were combined into a reaction mixture to achieve final concentrations of approximately 0.1 $\mu$M for each motor type and 1.5-2.5 $\mu$M for tubulin, referring to protein monomers for K401-micro and K401-iLID constructs, and protein dimers for tubulin. The sample preparation was conducted under dark-room conditions to minimize unintended light activation, using room light filtered to block wavelengths below 580 nm (Kodak Wratten Filter No. 25). The base reaction mixture included a buffer, MgATP as an energy source, glycerol as a crowding agent, pluronic F-127 for surface passivation, and components for oxygen scavenging (pyranose oxidase, glucose, catalase, Trolox, DTT), along with ATP-recycling reagents (pyruvate kinase/lactic dehydrogenase, phosphoenolpyruvic acid). The reaction mixture consisted of 59.2 mM K-PIPES pH 6.8, 4.7 mM MgCl2, 3.2 mM potassium chloride, 2.6 mM potassium phosphate, 0.74 mM EGTA, 1.4 mM MgATP, 10% glycerol, 0.50 mg/mL pluronic F-127, 2.9mg/mL pyranose oxidase, 3.2 mg/mL glucose, 0.086 mg/mL catalase, 5.4 mM DTT, 2.0 mM Trolox, 0.026 units/$\mu$L pyruvate kinase/lactic dehydrogenase, and 26.6 mM phosphoenolpyruvic acid.

We note that the sample is sensitive to the buffer pH and mixture incubation time. For our experimental conditions, the mixture pH is around 6.4 and we perform the experiments within 2 hours of constructing the mixture.

### Microscope setup

We conducted the experiments using an automated widefield epifluorescence microscope (Nikon Ti-2), custom-modified for two additional imaging modes: epi-illuminated pattern projection and LED-gated transmitted light. Light patterns from a programmable DLP chip (EKB Technologies DLP LightCrafter™ E4500 MKII™ Fiber Couple) were projected onto the sample via a user-modified epi-illumination attachment (Nikon T-FL). The DLP chip was illuminated by a fiber-coupled 470 nm LED (ThorLabs M470L3). The epi-illumination attachment featured two light-path entry ports: one for the projected pattern light path and the other for a standard widefield epi-fluorescence light path. These light paths were combined using a dichroic mirror (Semrock BLP01-488R-25). The magnification of the epi-illumination system was calibrated to ensure the camera's imaging sensor (FliR BFLY-U3-23S6M-C) was fully illuminated when the entire DLP chip was activated. Micro-Manager software, running custom scripts, controlled the pattern projection and stage movement. For the transmitted light path, we replaced the standard white-light brightfield source (Nikon T-DH) with an electronically time-gated 660 nm LED (ThorLabs M660L4-C5) to minimize light-induced linkage during brightfield imaging.

### Light pattern construction

In this work, we followed the illumination protocol from Ref. [7], using pulsed light to activate motor-linking. A light pattern is projected onto the active matter system every 10 seconds, with each pulse lasting 30 milliseconds. This protocol ensures sufficient motor activation for network contraction and sustained fluid flow. Shorter illumination periods fail to generate enough cross-linked microtubules, while longer or continuous illumination causes motor activation outside the intended region, disrupting the network and fluid flow.

For generating V-shape light patterns, we utilized a Python script to calculate the V shape's coordinates and mapped them onto a 1280 by 800 pixel canvas. The pattern was displayed as white on a black background. The final

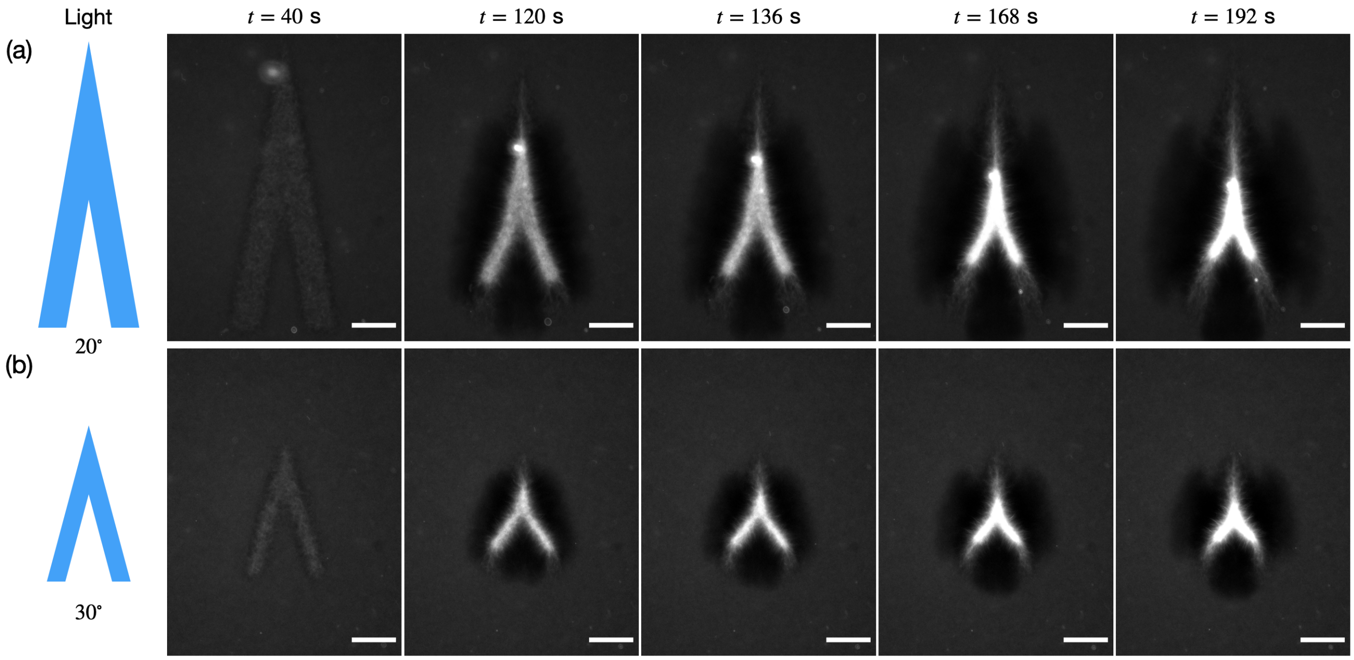


FIG. S6. Experimental images of concave networks with (a) $\theta = 20^\circ$ and (b) $\theta = 30^\circ$. Scale bars, 100 $\mu$m.

V shape pattern was saved as a TIFF file at the specified location for subsequent experimental use. A custom Java script was used to read the tiff file and project the custom light pattern to the active matter system in micro-manager.

### Image processing for centerline-curvature measurement

We first separated the left and right arms of the networks. We used the "skeletonize" function from the scikit-image package [23] in Python to extract the skeleton of each arm. We then used the FilFinder package [24] to pick out the longest branch of the skeleton, which is the centerline of the arm.

To measure the curvature of the centerline, we first fitted the centerline with a parabola $y = ax^2 + bx + c$. The local curvature $\kappa$ of the centerline is is $\kappa = y''/(1 + y'^2)^{3/2}$ and the average curvature $[\kappa]$ is

$$[\kappa] = \frac{\int_{x_0}^{x_t} y''/(1 + y'^2)\mathrm{d}x}{\int_{x_0}^{x_t} (1 + y'^2)^{1/2}\mathrm{d}x}, \tag{S36}$$

which can be numerically integrated using $y'' = 2a$ and $y' = 2ax + b$. $x_0$ and $x_t$ are the x-coordinates of the starting and ending points of the centerline, respectively.

### Experimental images of concave networks

We show more examples of concave V-shaped networks with small angles in Fig. S6.

* fy2@caltech.edu
† mthomson@caltech.edu
[1] P. W. Baas and S. Lin. Hooks and comets: The story of microtubule polarity orientation in the neuron. *Developmental Neurobiology*, 71:403–418, 2011.
[2] Z. He and Y. Jin. Intrinsic control of axon regeneration. *Neuron*, 90:437–451, 2016.

[3] M. Braun, D. R. Drummond, R. A. Cross, and A. D. McAinsh. The kinesin-14 Klp2 organizes microtubules into parallel bundles by an ATP-dependent sorting mechanism. *Nature Cell Biology*, 11:724–730, 2009.

[4] Laura Schaedel, Karin John, Jérémie Gaillard, Maxence V. Nachury, Laurent Blanchoin, and Manuel Théry. Microtubules self-repair in response to mechanical stress. *Nature Materials*, 14:1156–1163, 2015.

[5] Ken-Ichi Sano, Ryuzo Kawamura, Taiki Tominaga, Naoko Oda, Kuniharu Ijiro, and Yoshihito Osada. Self-repairing filamentous actin hydrogel with hierarchical structure. *Biomacromolecules*, 12:4173–4177, 2011.

[6] M. W. Elting, C. L. Hueschen, D. B. Udy, and S. Dumont. Force on spindle microtubule minus ends moves chromosomes. *J. Cell Biol.*, 206:245–256, 2014.

[7] Tyler D. Ross, Heun Jin Lee, Zijie Qu, Rachel A. Banks, Rob Phillips, and Matt Thomson. Controlling organization and forces in active matter through optically defined boundaries. *Nature*, 572:224–229, 2019.

[8] Fan Yang, Shichen Liu, Heun Jin Lee, Rob Phillips, and Matt Thomson. Dynamic flow control through active matter programming language. *Nature Materials*, 24:615–625, 2024.

[9] F. Nédélec, T. Surrey, and A. C. Maggs. Dynamic concentration of motors in microtubule arrays. *Phys. Rev. Lett.*, 86:3192–3195, 2001.

[10] T. Surrey, F. Nédélec, S. Leibler, and E. Karsenti. Physical properties determining self-organization of motors and microtubules. *Science*, 292:1167–1171, 2001.

[11] R. A. Banks, V. Galstyan, H. J. Lee, S. Hirokawa, A. Ierokomos, T. D. Ross, Z. Bryant, M. Thomson, and R. Phillips. Motor processivity and speed determine structure and dynamics of microtubule-motor assemblies. *Elife*, 12:e79402, 2023.

[12] B. Audoly and Y. Pomeau. *Elasticity and Geometry: From hair curls to the non-linear response of shells*. Oxford University Press, 2010.

[13] P. D. Howell, G. Kozyreff, and J. R. Ockendon. *Applied Solid Mechanics*. Cambridge University Press, 2009.

[14] F. Yang and H. A. Stone. Formation, rupture, and healing of an annular viscous film. *Phys. Rev. Lett.*, 124:224501, 2020.

[15] D. Oriola, F. Jülicher, and J. Brugués. Active forces shape the metaphase spindle through a mechanical instability. *Proc. Natl. Acad. Sci. U.S.A.*, 117(28):16154–16159, 2020.

[16] W. W. Ahmed, É. Fodor, M. Almonacid, M. Bussonnier, M. Verlhac, N. Gov, P. Visco, F. van Wijland, and T. Betz. Active mechanics reveal molecular-scale force kinetics in living oocytes. *Biophysical Journal*, 114:1667–1679, 2018.

[17] B. Najma, M. Varghese, L. Tsidilkovski, L. Lemma, A. Baskaran, and G. Duclos. Competing instatibilities reveal how to rationally design and control active crosslinked gels. *Nat. Commun.*, 13, 2022.

[18] S. Fürthauer, B. Lemma, P. J. Foster, S. C. Ems-McClung, C. H. Yu, C. E. Walczak, Z. Dogic, D. J. Needleman, and M. J. Shelley. Self-straining of actively crosslinked microtubule networks. *Nat. Phys.*, 15:1295–1300, 2019.

[19] S. Fürthauer, D. J. Needleman, and M. J. Shelley. A design framework for actively crosslinked filament networks. *New J. Phys.*, 2020.

[20] M.A. Alves, P.J. Oliveira, and F.T. Pinho. Numerical methods for viscoelastic fluid flows. *Annu. Rev. Fluid Mech.*, 53:509–541, 2021.

[21] L. G. Leal. *Advanced Transport Phenomena*. Cambridge University Press, 2007.

[22] G. Guntas, R. A. Hallett, S. P. Zimmerman, T. Williams, H. Yumerefendi, J. E. Bear, and B. Kuhlman. Engineering an improved light-induced dimer (iLID) for controlling the localization and activity of signaling proteins. *Proc. Natl. Acad. Sci. U. S. A.*, 112(1):112–117, 2015.

[23] S. van der Walt, J. L. Schönberger, J. Nunez-Iglesias, F. Boulogne, J. D. Warner, N. Yager, E. Gouillart, T. Yu, and the scikit-image contributors. Scikit-image: Image processing in Python. *PeerJ*, 2:e453, 2014.

[24] E. W. Koch and E. W. Rosolowsky. Filament identification through mathematical morphology. *Mon. Not. R. Astron. Soc.*, 452:3435–3450, 2015.
